# Efficacy of an Internet-based self-help intervention with human guidance or automated messages to alleviate loneliness: a three-armed randomized controlled trial

**DOI:** 10.1038/s41598-024-57254-0

**Published:** 2024-03-19

**Authors:** Noëmi Seewer, Andrej Skoko, Anton Käll, Gerhard Andersson, Maike Luhmann, Thomas Berger, Tobias Krieger

**Affiliations:** 1 Department of Clinical Psychology and Psychotherapy, University of Bern, 3012 Bern, Switzerland; 2 Department of Behavioral Sciences and Learning, and Biomedical and Clinical Sciences, Linköping University, 58183 Linköping, Sweden; 3 Department of Clinical Neuroscience, Karolinska Institute, 11763 Stockholm, Sweden; 4 Faculty of Psychology, Ruhr University Bochum, 44801 Bochum, Germany; 5 German Center for Mental Health (DZPG), 80336 Munich, Germany

**Keywords:** Psychology, Psychiatric disorders

## Abstract

Loneliness is a prevalent and stigmatized phenomenon associated with adverse (mental) health outcomes. However, evidence-based interventions to alleviate loneliness are scarce. This randomized controlled trial (ClinicalTrials.gov-ID: NCT04655196) evaluated the efficacy of an internet-based cognitive behavioral self-help intervention (ICBT) to reduce loneliness by comparing two intervention groups with guidance or automated messages against a waitlist control group. Adults (*N* = 243) suffering from loneliness were recruited from the general public and then randomly assigned (2:2:1) to a 10-week ICBT with human guidance (GU) or automated messages (AM) or to a waitlist control group (WL). Loneliness, assessed with the UCLA-9, was the primary outcome. Outcomes were assessed at baseline and 10 weeks (post) and analyzed using mixed-effects models. The pooled intervention conditions resulted in lower loneliness scores at post-assessment than the WL (Cohen’s *d* = 0.57, 95% CI [0.25; 0.89]) and reduced depressive symptoms, social anxiety, social avoidance behavior, and rejection sensitivity (*d* = 0.32–0.52). The GU group had lower loneliness scores at post-assessment than the AM group (*d* = 0.42, 95% CI [0.13; 0.70]). ICBT effectively alleviated loneliness, and guidance increased the reduction in loneliness compared to automated messages. Alleviating loneliness with ICBT further seems to reduce the overall burden of psychopathological symptoms.

## Introduction

Loneliness arises when fundamental needs for human connections are not met^1^. It can be defined as an aversive subjective experience resulting from a discrepancy between actual and desired social relationships in terms of their quality and/or quantity^2^. One person can feel lonely despite being surrounded by people, while another person with a small social network may not. Thus, despite being related, loneliness and objective social isolation, i.e., lack of a social network, only show small correlations^3^. Albeit prevalence rates of loneliness have increased after the beginning of the COVID-19 pandemic^4^, loneliness was even before a prevalent phenomenon. Between 2007 and 2012, in the German general population, around 10% reported feelings of loneliness^5^, and a meta-analysis including studies from high-income countries published between 2008 and 2020 implies that around one in four of the older adult population feels lonely at least sometimes and 7.9% reported severe loneliness^6^.

Loneliness seems not restricted to a specific age group but is prevalent across the lifespan^7–9^. Moreover, evidence suggests loneliness to be associated with adverse (mental) health outcomes, e.g., cardiovascular and brain health^10^, depression^11^, social anxiety^12^, suicidal ideation and behavior^13^, overall well-being^14^, and an increased risk for early mortality, even after controlling for confounding variables such as depression^15,16^. Consequently, loneliness is increasingly recognized as a major public health concern^17^. Therefore, evidence-based interventions are needed to alleviate the individual and societal burden of chronic loneliness efficiently.

From an evolutionary perspective, loneliness is a driving force in maintaining existing and forming new social relationships to increase the chance of survival^18^. Thus, transient feelings of loneliness are a common and adaptive human experience. However, for some individuals, loneliness persists over a prolonged period and may have lost its adaptive characteristics^18^. A cognitive model of chronic loneliness^19,20^ was proposed to describe the development and maintenance of chronic loneliness. It is assumed that feelings of loneliness trigger hypervigilance for potentially threatening stimuli in social situations leading to cognitive biases in social information processing, e.g., negative evaluation of the self and others. As a result, lonely people show behaviors, e.g., social withdrawal or passivity, that prevent them from gaining positive experiences in social situations. Because of this, feelings of loneliness persist through a self-perpetuating vicious cycle. Notably, negative associations with adverse health outcomes are predominantly reported in individuals experiencing chronic loneliness^21^.

In line with a cognitive model of loneliness^20^, previous meta-analyses have suggested that interventions aimed at changing maladaptive social cognitions are the most effective in reducing loneliness and promoting social connectedness^22,23^. Findings of a recent meta-analysis corroborated these results in showing the efficacy of psychological interventions in alleviating loneliness, with cognitive behavioral interventions belonging to the most efficacious ways of reducing loneliness—however, not superior to other psychological interventions^24^. Hickin and colleagues^24^ further stress the need for more high-quality studies on the efficacy of loneliness interventions.

Despite the availability of evidence-based treatments for various mental health problems, a treatment gap still hinders many people from accessing those treatments^25^. Technological advances have allowed psychological interventions to be delivered via the Internet and thus potentially reach more people needing treatment^26^. Evidence-based treatment manuals based on the face-to-face literature have often been adapted for the online setting and delivered as so-called Internet-based self-help programs. Many internet-delivered programs are based on cognitive behavioral therapy (CBT) principles and are often named ICBT. The interventions frequently consist of several modules that can be worked on independently by the users. ICBT has proven effective across multiple psychiatric and somatic disorders^26^, and ICBT with guidance demonstrated comparable effectiveness to face-to-face therapies^27^. Due to the time and place-independent accessibility, scalability, and anonymity^26^, Internet-based self-help interventions can reach more people in need of psychological treatment, especially those with (self-) stigmatized conditions such as loneliness^28^.

Internet-based interventions to reduce loneliness have been developed and tested in initial studies and shown promising results^29,30^. In a pilot RCT, guided ICBT was compared to a waitlist control group^29^. Loneliness was significantly reduced after the intervention phase with a between-group effect size of *d* = 0.77. Further support for the efficacy of ICBT was reported in a three-armed trial comparing ICBT against an Internet-based Interpersonal Therapy intervention (IIPT)^30^. While loneliness was significantly reduced in participants in the ICBT condition compared to the waitlist control condition (Cohen’s *d* = 0.71) and the IIPT condition (Cohen’s *d* = 0.53) at post-assessment, no statistically significant difference was found between IIPT and the waitlist condition. While the findings of these studies highlight that loneliness can effectively be reduced with guided ICBT, the study designs do not allow controlling for the effects of guidance (i.e., weekly feedback by a therapist or coach). However, this could be relevant, especially in the context of loneliness, as human guidance could touch upon aspects relevant to satisfying social relationships (e.g., validation) and thus lead to a greater reduction in loneliness^29^.

A further study examined an unguided Internet-based friendship enrichment program to reduce loneliness^31^. In this trial, only one third of the participants completed all modules^32^. Studies have found improved treatment completion rates when an automated email message reminds patients to continue working on the treatment^33^. Furthermore, it has been shown that Internet-based interventions with human guidance can increase adherence to the intervention, i.e., raise the average amount of intervention completion (*g* = 0.29, 95% CI [0.18; 0.40])^34^ and lead to greater effects than unguided (human guidance: *g* = 0.63, 95% CI [0.50; 0.76]; unguided: *g* = 0.34, 95% CI [0.24; 0.45])^35^ or technologically guided interventions, i.e., automated messages (pooled effect size in favor of human guidance: *g* = 0.11, 95% CI [0.03; 0.19])^36^. In a more recent meta-analysis, a higher degree of human contact was associated with a better treatment outcome after internet-based interventions for depression, i.e. contact before and during treatment: Standardized mean difference (SMD) = 0.57, 95% CI [0.44; 0.71] versus contact before treatment only: SMD = 0.48, 95% CI [0.33; 0.63])^37^. It is thus of relevance to further examine the effects of human guidance on the outcome of Internet-based interventions for loneliness.

The present study was designed to examine the effects of an ICBT program against loneliness. The ICBT program addressed aspects relevant to the cognitive model of loneliness described above (e.g., maladaptive social cognitions, avoidance behavior) to break the vicious cycle of loneliness (see “Methods” section for a more detailed description of the ICBT program). In a three-armed randomized controlled trial (RCT), we compared ICBT with human guidance and ICBT with automated messages against a waitlist control group. Additionally, we compared the intervention groups against each other to investigate the added effect of human guidance. Our primary hypothesis was that participants in the pooled intervention conditions would show greater reductions in loneliness and secondary outcomes such as depressive symptoms, social anxiety symptoms, and cognitive bias compared to the waitlist control group. Additionally, we expected participants in the guided condition to show greater improvements regarding loneliness and more favorable results on the secondary outcomes than in the automated message condition.

## Results

### Baseline comparisons and preliminary analyses

Table 1 shows the baseline characteristics of the full sample. There were no significant baseline differences between groups on any demographic and loneliness-specific variables, nor regarding psychopathology (all *p*’s > 0.07). Supplementary Table S2 shows baseline values and differences between groups on the primary and secondary outcomes. Participants did not significantly differ on any measure (all *p*’s > 0.26), except for the DDI (*F*(2,239) = 3.38, *p* = 0.04, *η*^2^ = 0.03), with significantly higher scores in the GU compared to the AM condition (*p* = 0.04). Regarding loneliness, participants had a mean value of 7.56 (*SD* = 2.14) on the 3-item short form of the UCLA Loneliness Scale. This means that a total of 241 (99.2%) participants had higher scores than the norm sample from the German general population, with 164 (67.5%) participants presenting higher scores than 95% of the German general population^38^.

**Table 1:** Baseline characteristics ITT-sample.

	GU (*n* = 98)	AM (*n* = 97)	WL (*n* = 48)	Statistic
Mean age, years (SD)	46.2 (15.5)	45.6 (14.7)	45.2 (14.1)	*F* (2,240) = 0.09; *p* = 0.92
Gender, n (%)				*χ*^2^(4) = 1.91; *p* = 0.75
Female	76 (77.6%)	77 (79.4%)	38 (79.2%)	
Male	21 (21.4%)	20 (20.6%)	9 (18.8%)	
Other	1 (1.0%)	0 (0.0%)	1 (2.1%)	
Marital status n (%)				*χ*^2^(2) = 2.11; *p* = 0.35
Single/divorced/widowed	72 (73.5%)	77 (79.4%)	33 (68.8%)	
Married/partnered	26 (26.5%)	20 (20.6%)	15 (31.2%)	
Living situation, *n* (%)				*χ*^2^(6) = 5.57; *p* = 0.47
Alone	66 (67.3%)	63 (64.9%)	24 (50.0%)	
With partner/family	17 (17.3%)	20 (20.6%)	12 (25.0%)	
Shared flat	10 (10.2%)	10 (10.3%)	7 (14.6%)	
Other	5 (5.1%)	4 (4.1%)	5 (10.4%)	
Highest educational level, *n* (%)				*χ*^2^(4) = 4.36; *p* = 0.36
Middle school	4 (4.1%)	0 (0.0%)	1 (2.1%)	
High school/some college	32 (33.0%)	37 (38.1%)	17 (35.4%)	
University degree	61 (62.9%)	60 (61.9%)	30 (62.5%)	
Employment, *n* (%)				*χ*^2^(10) = 17.23; *p* = 0.07
Full-time paid work	32 (32.7%)	32 (33.7%)	16 (34.0%)	
Part-time paid work	28 (28.6%)	27 (28.4%)	17 (36.2%)	
Student/in training	7 (7.1%)	5 (5.3%)	3 (6.4%)	
Unemployed	3 (3.1%)	12 (12.6%)	3 (6.4%)	
Househusband/Housewife	2 (2.0%)	2 (2.1%)	4 (8.5%)	
Retired	26 (26.5%)	17 (17.9%)	4 (8.5%)	
Current psychological treatment^a^	31 (31.6%)	33 (34.0%)	15 (31.3%)	*χ*^2^(2) = 0.17; *p* = 0.92
Current use of psychotropic medication^a^	13 (13.3%)	17 (17.5%)	10 (20.8%)	*χ*^2^(2) = 1.48; *p* = 0.48
Mean duration of loneliness, months (*SD*)^b^	128.42 (153.60)	117.38 (137.55)	206.17 (224.05)	Kruskal–Wallis *χ*^2^(2) = 4.89; *p* = 0.09
Psychiatric diagnoses^c^				
Major depressive disorder	9 (9.2%)	13 (13.4%)	5 (10.4%)	*χ*^2^(2) = 0.91; *p* = 0.64
Panic disorder	5 (5.1%)	7 (7.2%)	5 (10.4%)	*χ*^2^(2) = 1.41; *p* = 0.49
Agoraphobia	2 (2.0%)	7 (7.2%)	5 (10.4%)	*χ*^2^(2) = 4.79; *p* = 0.09
Social anxiety disorder	25 (25.5%)	29 (29.9%)	17 (35.4%)	*χ*^2^(2) = 1.56; *p* = 0.45
Generalized anxiety disorder	17 (17.3%)	14 (14.4%)	7 (14.6%)	*χ*^2^(2) = 0.36; *p* = 0.83
Obsessive compulsive disorder	3 (3.1%)	4 (4.1%)	1 (2.1%)	*χ*^2^(2) = 0.48; *p* = 0.80
Post-traumatic stress disorder	2 (2.0%)	5 (5.2%)	3 (6.3%)	*χ*^2^(2) = 1.89; *p* = 0.39
Eating disorder	4 (4.1%)	2 (2.1%)	1 (2.1%)	*χ*^2^(2) = 0.85; *p* = 0.65

*GU* SOLUS-D with guidance, *AM* SOLUS-D with automated message, *WL* waitlist control group.

^a^Reflects the number and percentage of participants answering “yes” to this question.

^b^GU: n = 97; AM: n = 94; WL: n = 47.

^c^Reflects the number and percentage of participants fulfilling the respective psychological diagnosis as indicated by the Mini-DIPS during screening.

### Study dropout analysis

In total, 63 participants (25.9%; GU, *n* = 28; AM, *n* = 33; WL, *n* = 2) did not complete the questionnaires at post-assessment. Non-completers did not differ from completers regarding primary or secondary outcomes at baseline (all *p*’s > 0.06), nor demographic variables (*p*’s > 0.10), except for age, where non-completers were significantly younger, (*t*(241) = − 2.62, *p* = 0.009) (see Supplementary Tables S3 and S4). Additionally, non-completion rates significantly differed between study conditions, *χ*^2^(2, *n* = 243) = 15.50, *p* < 0.001, *V* = 0.25), with a higher number of non-completers in the intervention conditions compared to the WL (GU vs. WL: *χ*^2^(1, *n* = 146) = 11.75, *p* < 0.001, *V* = 0.28; AM vs. WL: *χ*^2^(1, *n* = 145) = 15.63, *p* < 0.001, *V* = 0.33).

### Intervention Usage

Data on the use of SOLUS-D was not available for five (2.6%) participants, as one (0.5%) deleted their account, one (0.5%) was not able to access the intervention due to technical problems, and three (1.5%) had never logged into the program after randomization without indicating reasons. Nevertheless, these participants were included in the descriptive statistics regarding the intervention usage. In total, 42 (42.9%) participants in the GU and 39 (40.2%) in the AM condition accessed all nine modules during the intervention phase. A total of 84.7% (*n* = 83) in the GU condition accessed at least four modules (number of modules for minimal therapeutic contact) compared to 72.2% (*n* = 70) in the AM condition (Thanks to a reviewer’s suggestion, we further conducted a chi-squared test to examine if the number of participants who accessed 4 or more modules differed between both intervention conditions. Despite a statistically significant chi-squared test, *χ*^2^(1) = 4.53, *p* = 0.03, *V* = 0.15), post-hoc analyses comparing residuals to a critical value yielded no significant results). On average, participants in the GU condition accessed 6.77 (*SD* = 2.62, *Md* = 8) out of nine modules, while participants in the AM condition accessed 6.07 (*SD* = 3.16, *Md* = 7) modules. The two groups did not significantly differ in their mean number of modules accessed (*t*(186) = − 1.67, *p* = 0.10). Participants in the GU condition spent on average 563.28 min (*SD* = 543.86, *Md* = 451) in the program compared to the AM condition with an average time of 370.44 min (*SD* = 338.36, *Md* = 292), which indicates a significant difference (*t*(193) = − 2.97, *p* = 0.003, *d* = − 0.43).

### Intervention effects on primary outcome

**Figure 1:**
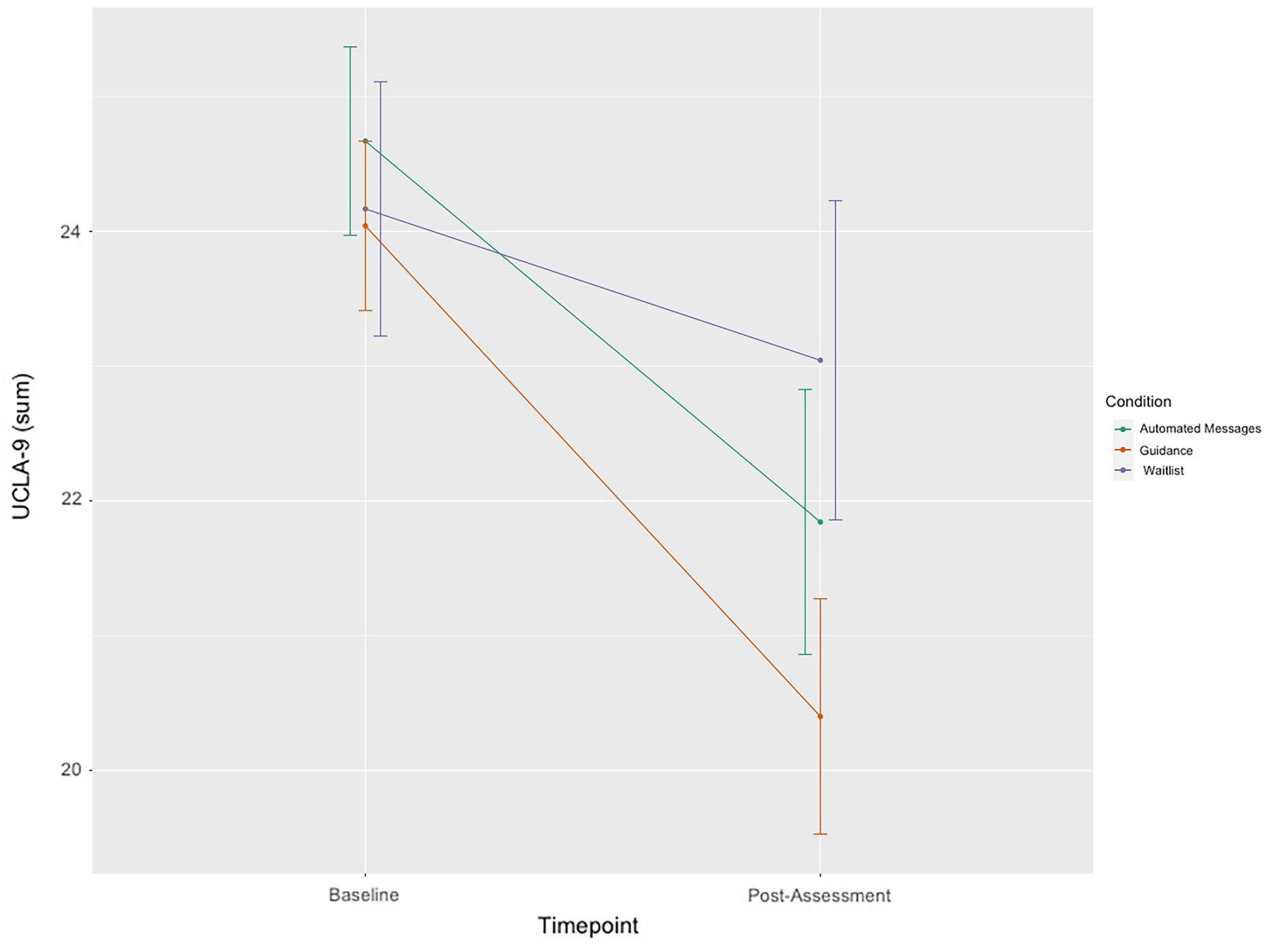
Observed means on the University of California Loneliness Scale – 9-item short form (UCLA-9) at both time points with 95% confidence intervals.

Observed means for the primary outcome measure UCLA-9 for GU, AM, and WL at baseline, and 10 weeks are presented in Fig. 1.

Observed and estimated means on the primary outcome assessed at baseline and post-assessment are displayed in Table 2. Table 3 shows effect sizes for within- and between-group differences, overall effects, and contrasts for significant Time × Group interactions. Regarding the primary outcome, a linear mixed model showed a significant Time × Group interaction, *F*(2,191.98) = 8.22, *p* < 0.001 (see Supplementary Table S5 for detailed results of the mixed effects model). Subsequent planned contrast analyses revealed significantly lower loneliness scores at post-assessment for the intervention conditions compared to the WL (*t*(241) = 3.13, *p* < 0.002, *d* = 0.57, 95% CI [0.25; 0.89]), with an additional significant difference between both intervention conditions in favor of the GU condition (*t*(193) = 2.38, *p* = 0.02, *d* = 0.42, 95% CI [0.13; 0.70]) (see Supplementary Table S22 for a summary of the contrast analyses). Additionally, medium to large statistically significant within-group effects were found for the intervention groups (GU, *d* = 1.02, 95% CI [0.71; 1.31]; AM, *d* = 0.73, 95% CI [0.43; 1.02]) and a small, non-significant effect for the WL (*d* = 0.28, 95% CI [− 0.12; 0.68]).

**Table 2:** Observed and estimated means for primary and secondary outcome measures.

Measure	Condition	Baseline		Post (observed)		Post (estimated)	
		Mean (*SD*)	*n*	Mean (*SD*)	*n*	Mean (*SE*)	*n*
UCLA-9	GU	24.04 (3.18)	98	20.40 (3.73)	70	20.53 (0.41)	98
	AM	24.67 (3.51)	97	21.84 (4.01)	64	21.93 (0.42)	97
	WL	24.17 (3.33)	48	23.04 (4.10)	46	23.12 (0.53)	48
PHQ-9	GU	8.90 (3.35)	98	6.12 (3.11)	69	6.00 (0.41)	98
	AM	8.87 (3.31)	97	6.67 (3.31)	64	6.87 (0.42)	97
	WL	8.50 (3.24)	48	8.20(4.94)	46	8.15 (0.51)	48
SIAS-6	GU	5.73 (4.36)	98	4.26 (3.38)	66	4.64 (0.47)	98
	AM	5.85 (4.73)	96	4.73 (3.45)	59	4.67 (0.48)	96
	WL	5.81 (3.93)	48	5.82 (4.66)	44	5.69 (0.63)	48
SPS-6	GU	3.23 (3.28)	98	2.77 (3.05)	66	2.78 (0.41)	98
	AM	3.42 (4.22)	96	2.17 (2.74)	59	2.27 (0.42)	96
	WL	3.25 (3.64)	48	4.02 (4.89)	44	3.93 (0.54)	48
SNI	GU	10.63 (5.24)	98	11.14 (5.23)	70	11.11 (0.71)	98
	AM	11.33 (7.05)	97	11.55 (7.01)	64	11.74 (0.73)	97
	WL	11.50 (6.40)	48	12.11 (6.96)	46	12.02 (0.92)	48
RSES	GU	1.81 (0.65)	98	2.09 (0.55)	66	2.08 (0.07)	98
	AM	1.74 (0.70)	96	2.10 (0.58)	59	2.02 (0.08)	96
	WL	1.69 (0.72)	48	1.84 (0.64)	44	1.86 (0.10)	48
SWLS	GU	19.18 (5.93)	98	20.24 (6.37)	66	20.39 (0.70)	98
	AM	17.85 (5.97)	97	20.17 (6.52)	59	19.91 (0.72)	97
	WL	17.88 (7.19)	48	18.84 (6.98)	44	18.78 (0.94)	48
SOCS-S	GU	3.39 (0.64)	98	3.65 (0.59)	68	3.65 (0.07)	98
	AM	3.36 (0.66)	97	3.52 (0.70)	61	3.45 (0.08)	97
	WL	3.42 (0.65)	48	3.36 (0.57)	45	3.37 (0.09)	48
CBAS	GU	2.44 (0.75)	98	2.30 (0.61)	67	2.30 (0.08)	98
	AM	2.50 (0.76)	97	2.41 (0.67)	60	2.40 (0.08)	97
	WL	2.47 (0.80)	48	2.63 (0.82)	43	2.60 (0.11)	48
IJQ_tot	GU	1.61 (0.40)	97	1.45 (0.43)	64	1.47 (0.05)	97
	AM	1.65 (0.41)	91	1.46 (0.53)	53	1.50 (0.06)	91
	WL	1.73 (0.55)	47	1.67 (0.53)	44	1.67 (0.07)	47
DDI	GU	3.26 (0.82)	97	3.45 (0.69)	67	3.49 (0.09)	97
	AM	2.96 (0.82)	97	3.16 (0.76)	60	3.20 (0.09)	97
	WL	3.00 (0.91)	48	3.01 (0.83)	43	3.04 (0.12)	48
PID5BF+	GU	1.03 (0.31)	98	0.82 (0.32)	66	0.86 (0.04)	98
	AM	1.07 (0.35)	96	0.96 (0.33)	59	0.97 (0.04)	96
	WL	1.03 (0.31)	48	0.95 (0.35)	43	0.95 (0.05)	48
BVI	GU	1.64 (1.00)	98	1.44 (1.05)	66	1.42 (0.11)	98
	AM	1.79 (0.99)	97	1.54 (0.96)	59	1.53 (0.11)	97
	WL	1.68 (0.90)	48	1.67 (0.97)	44	1.70 (0.14)	48
KGAI-SF	GU	3.59 (0.53)	98	3.78 (0.49)	66	3.76 (0.06)	98
	AM	3.49 (0.59)	97	3.69 (0.56)	60	3.63 (0.06)	97
	WL	3.55 (0.50)	48	3.59 (0.47)	44	3.60 (0.08)	48
A-RSQ	GU	11.52 (4.00)	97	9.45 (4.02)	66	9.80 (0.48)	97
	AM	11.21 (4.38)	97	9.53 (4.26)	60	9.73 (0.50)	97
	WL	10.99 (4.34)	48	11.47 (5.06)	44	11.36 (0.63)	48
MSS-SF	GU	2.43 (0.64)	98	2.67 (0.65)	68	2.68 (0.07)	98
	AM	2.36 (0.66)	97	2.50 (0.67)	62	2.50 (0.08)	97
	WL	2.48 (0.59)	48	2.55 (0.66)	46	2.55 (0.09)	48
Lonely_dir	GU	1.88 (0.69)	98	1.13 (0.60)	68	1.17 (0.08)	98
	AM	1.95 (0.75)	96	1.27 (0.61)	62	1.32 (0.08)	96
	WL	1.98 (0.76)	48	1.57 (0.72)	46	1.57 (0.10)	48

*UCLA-9* 9-item version of the UCLA Loneliness Scale, *PHQ-9* 9-item Depression Module of the Patient Health Questionnaire, *SIAS-6* Social Interaction Anxiety Scale, *SPS-6* Social Phobia Scale, *SNI* Social Network Index—size of social network, *SWLS* satisfaction with life, *RSES* Rosenberg self-esteem Scale; *SOCS-S* Sussex-Oxford Compassion for the Self Scale, *CBAS* Cognitive-Behavioral Avoidance Scale—subscale Behavior-social avoidance, *IJQ_tot* Interpretation and Judgmental Bias Questionnaire—total score, *DDI* Distress Disclosure Index, *PID5BF*+ Personality Inventory for the DSM-5 Brief Form Plus, *BVI* Bern Embitterment Inventory—subscale misanthropy, *KGAI-SF* Kernis Goldman Authenticity Inventory-short form, *A-RSQ* Adult-Rejection Sensitivity Questionnaire, *MSS-SF* the Motivation for Solitude Scale – Short Form, *Lonely_dir* single item to assess loneliness directly (“Do you feel lonely?”), *GU* SOLUS-D with guidance, *AM* SOLUS-D with automated message, *WL* waitlist control group.

**Table 3:** Within- and between-group effect sizes, overall effects, and contrasts at post-assessment for primary and secondary outcome measures.

Measure	Condition	Pre–post within-group effect sizes (estimated means)		Overall effects (Time × Group interaction)	Contrasts (at post-assessment)	Between-group effect sizes at post-treatment (estimated means)
		Cohen’s d [95% CI]	n	F and *df*		Cohen’s *d* [95% CI]
UCLA-9	GU	1.02 [0.71; 1.31]	98	*F*_(2,191.98)_ = 8.22 *p* < 0.001	WL vs. INT: *p* = 0.002 GU vs. AM: *p* = 0.02	GU vs. WL: − 0.80 [− 1.16; − 0.44]
	AM	0.73 [0.43; 1.02]	97			GU vs. AM: − 0.42 [− 0.70; − 0.13]
	WL	0.28 [− 0.12; 0.68]	48			AM vs. WL: − 0.34 [− 0.60; 0.01]
PHQ-9	GU	0.89 [0.60; 1.18]	98	*F*_(2,199.83)_ = 6.91 *p* = 0.001	WL vs. INT: *p* = 0.004 GU vs. AM: *p* = 0.14	GU vs. WL: − 0.65 [− 1.00; − 0.29]
	AM	0.60 [0.31; 0.89]	97			GU vs. AM: − 0.26 [− 0.54; 0.02]
	WL	0.08 [− 0.32; 0.48]	48			AM vs. WL: − 0.39 [− 0.73; − 0.04]
SIAS-6	GU	0.28 [0.00; 0.56]	98	*F*_(2,169.24)_ = 2.35 *p* = 0.10	–	GU vs. WL: − 0.25 [− 0.59; 0.10]
	AM	0.29 [0.00; 0.57]	96			GU vs. AM: − 0.01 [− 0.29; 0.27]
	WL	0.03 [− 0.37; 0.43]	48			AM vs. WL: − 0.23 [− 0.57; 0.12]
SPS-6	GU	0.14 [− 0.14; 0.42]	98	*F*_(2,174.71)_ = 7.10 *p* = 0.001	WL vs. INT: *p* = 0.02 GU vs. AM: *p* = 0.38	GU vs. WL: − 0.34 [− 0.69; 0.01]
	AM	0.32 [0.04; 0.61]	96			GU vs. AM: 0.13 [− 0.15; 0.42]
	WL	− 0.16 [− 0.56; 0.24]	48			AM vs. WL: − 0.41 [− 0.76; − 0.06]
SNI	GU	− 0.09 [− 0.37; 0.19]	98	*F*_(2,188.65)_ = 0.007 *p* = 0.99	–	GU vs. WL: − 0.16 [− 0.51; 0.18]
	AM	− 0.06 [− 0.34; 0.22]	97			GU vs. AM: − 0.10 [− 0.38; 0.18]
	WL	− 0.08 [− 0.48; 0.32]	48			AM vs. WL: − 0.04 [− 0.39; 0.30]
RSES	GU	− 0.46 [− 0.74; − 0.17]	98	*F*_(2,178.36)_ = 0.65 *p* = .52	–	GU vs. WL: 0.33 [− 0.02; 0.68]
	AM	− 0.44 [− 0.72; − 0.15]	96			GU vs. AM: 0.09 [− 0.19; 0.37]
SWLS	GU	− 0.20 [− 0.48; 0.09]	98	*F*_(2,172.48)_ = 1.24 *p* = 0.29	–	GU vs. WL: 0.24 [− 0.04; 0.52]
	AM	− 0.33 [− 0.61; − 0.05]	97			GU vs. AM: 0.08 [− 0.20; 0.36]
	WL	− 0.13 [− 0.53; 0.27]	48			AM vs. WL: 0.17 [− 0.11; 0.45]
SOCS-S	GU	− 0.43 [− 0.71; − 0.14]	98	*F*_(2,185.17)_ = 5.06 *p* = 0.007	WL vs. INT: *p* = 0.09 GU vs. AM: *p* = 0.05	GU vs. WL: 0.44 [0.09; 0.79]
	AM	− 0.13 [− 0.41; 0.15]	97			GU vs. AM: 0.31 [0.03; 0.59]
	WL	0.08 [− 0.32; 0.48]	48			AM vs. WL: 0.13 [− 0.22; 0.47]
CBAS	GU	0.20 [− 0.08; 0.48]	98	*F*_(2,173.67)_ = 4.26 *p* = 0.02	WL vs. INT: *p* = 0.05 GU vs. AM: *p* = .40	GU vs. WL: − 0.39 [− 0.73; − 0.04]
	AM	0.15 [− 0.14; 0.43]	97			GU vs. AM: − 0.13 [− 0.41; 0.15]
	WL	− 0.16 [− 0.56; 0.24]	48			AM vs. WL: − 0.25 [− 0.60; 0.09]
IJQ_tot	GU	0.33 [0.04; 0.61]	97	*F*_(2,176.71)_ = 0.84 *p* = 0.43	–	GU vs. WL: − 0.43 [− 0.78; − 0.08]
	AM	0.32 [0.03; 0.61]	91			GU vs. AM: − 0.06 [− 0.35; 0.22]
	WL	0.11 [− 0.29; 0.52]	47			AM vs. WL: − 0.37 [− 0.72; − 0.01]
DDI	GU	− 0.31 [− 0.59; − 0.02]	97	*F*_(2,172.29)_ = 2.55 *p* = 0.08	–	GU vs. WL: 0.53 [0.18; 0.88]
	AM	− 0.29 [− 0.57; − 0.01]	97			GU vs. AM: 0.36 [0.08; 0.64]
	WL	− 0.05 [− 0.45; 0.35]	48			AM vs. WL: 0.18 [− 0.16; 0.53]
PID5BF+	GU	0.54 [0.25; 0.82]	98	*F*_(2,171.04)_ = 2.34 *p* =0 .10	–	GU vs. WL: − 0.30 [− 0.63; 0.06]
	AM	0.28 [0.00; 0.56]	96			GU vs. AM: − 0.34 [− 0.63; − 0.06]
	WL	0.25 [− 0.15; 0.65]	48			AM vs. WL: 0.06 [− 0.29; 0.40]
BVI	GU	0.22 [− 0.07; 0.50]	98	*F*_(2,173.40)_ = 3.07 *p* = 0.05	WL vs. INT: *p* = 0.18 GU vs. AM: *p* = 0.46	GU vs. WL: − 0.29 [− 0.64; 0.06]
	AM	0.27 [− 0.02; 0.55]	97			GU vs. AM: − 0.12 [− 0.40; 0.17]
	WL	− 0.02 [− 0.42; 0.38]	48			AM vs. WL: − 0.17 [− 0.52; 0.17]
KGAI-SF	GU	− 0.35 [− 0.63; − 0.07]	98	*F*_(2,174.09)_ = 1.51 *p* = 0.22	−	GU vs. WL: 0.31 [− 0.03; 0.66]
	AM	− 0.25 [− 0.53; 0.03]	97			GU vs. AM: 0.24 [− 0.04; 0.52]
	WL	− 0.10 [− 0.50; 0.30]	48			AM vs. WL: 0.06 [− 0.29; 0.40]
A-RSQ	GU	0.42 [0.14; 0.71]	97	*F*_(2,177.15)_ = 6.89 *p* = 0.001	WL vs. INT: *p* = 0.03 GU vs. AM: *p* = 0.92	GU vs. WL: − 0.38 [− 0.73; − 0.03]
	AM	0.34 [0.06; 0.62]	97			GU vs. AM: 0.02 [− 0.27; 0.30]
	WL	− 0.08 [− 0.48; 0.32]	48			AM vs. WL: − 0.37 [− 0.72; − 0.02]
MSS-SF	GU	− 0.40 [− 0.68; − 0.12]	98	*F*_(2,188.96)_ = 2.00 *p* = 0.14	–	GU vs. WL: 0.23 [− 0.06; 0.51]
	AM	− 0.21 [− 0.49; 0.07]	97			GU vs. AM: 0.28 [0.00; 0.56]
	WL	− 0.11 [− 0.51; 0.29]	48			AM vs. WL: − 0.07 [− 0.35; 0.21]
Lonely_dir	GU	1.10 [0.79; 1.39]	98	*F*_(2,185.70)_ = 2.70 *p* = 0.07	−	GU vs. WL: − 0.55 [− 0.91; − 0.20]
	AM	0.92 [0.62; 1.21]	96			GU vs. AM: − 0.21 [− 0.50; 0.07]
	WL	0.56 [0.15; 0.96]	48			AM vs. WL: − 0.32 [− 0.67; − 0.03]

*Notes. UCLA-9* 9-item version of the UCLA Loneliness Scale, *PHQ-9* 9-item Depression Module of the Patient Health Questionnaire, *SIAS-6* Social Interaction Anxiety Scale, *SPS-6* Social Phobia Scale, *SNI* Social Network Index—size of social network, *SWLS* satisfaction with life, *RSES* Rosenberg self-esteem Scale; *SOCS-S* Sussex-Oxford Compassion for the Self Scale, *CBAS* Cognitive-Behavioral Avoidance Scale—subscale Behavior-social avoidance, *IJQ_tot* Interpretation and Judgmental Bias Questionnaire—total score, *DDI* Distress Disclosure Index, *PID5BF*+ Personality Inventory for the DSM-5 Brief Form Plus, *BVI* Bern Embitterment Inventory—subscale misanthropy; *KGAI-SF* Kernis Goldman Authenticity Inventory—short form, *A-RSQ* Adult-Rejection Sensitivity Questionnaire, *MSS-SF* the Motivation for Solitude Scale-Short Form, *Lonely_dir* single item to assess loneliness directly (“Do you feel lonely?”), *GU* SOLUS-D with guidance, *AM* SOLUS-D with automated message, *WL* waitlist control group, *INT* SOLUS-D with guidance and SOLUS-D with automated message taken together.

### Intervention effects on secondary outcomes

In terms of secondary outcomes (see Tables 2 and 3), significant Time × Group interactions were found for depressive symptoms, social anxiety symptoms, self-compassion, social avoidance behavior, misanthropy, and rejection sensitivity (*p’*s = 0.001–0.05) (see Supplementary Tables S6–S21 for detailed results of the mixed effects models). Consecutive contrast analyses comparing both intervention groups to the waitlist control group showed significantly lower depressive symptoms (PHQ-9; *t*(241) = 2.89, *p* = 0.004, *d* = 0.52), social anxiety symptoms (SPS-6; *t*(240) = 2.30, *p* = 0.02, *d* = 0.37), social avoidance behavior (CBAS; *t*(241) = 1.98, *p* = 0.048, *d* = 0.32), and lower rejection sensitivity (A-RSQ; *t*(240) = 2.22, *p* = 0.03, *d* = 0.38) at post-assessment in favor of the intervention groups. See Supplementary Table S22 for a summary of the contrast analyses. For the other secondary outcomes with significant Time × Group interactions, i.e., self-compassion (SOCS-S), and misanthropy (BVI), contrast analyses comparing both intervention groups with the waitlist control group did not show significant differences at post-assessment (*p*’s ranging from 0.09 to 0.18, *d*’s ranging from 0.23 to 0.29). Contrast analyses comparing both intervention conditions against each other did not show significant differences at post-assessment on any secondary outcome (*p*’s ranging from 0.05 to 0.92, *d*’s from 0.02 to 0.31).

Focusing on change within groups, significant pre-post effects in the GU condition ranged from small to large (0.28 [SIAS-6] to 1.10 [Lonely_dir]) and were headed in the expected direction. No significant within-group effects were found regarding social anxiety symptoms (SPS-6), objective social isolation, satisfaction with life, social avoidance behavior, and misanthropy for the GU condition. For the AM condition, significant within-group effects ranged from small to large (0.28 [PID5BF+] to 0.92 [Lonely_dir]). Within this group, no significant pre-post effects were found for objective social isolation, self-compassion, social avoidance behavior, misanthropy, authenticity, and motivation for solitude. The waitlist control condition did not improve significantly on any of the secondary measures, except for the single-item question assessing loneliness directly (*d* = 0.56; 95% CI [0.15; 0.96]), corresponding to a reduction in loneliness.

### Sensitivity analyses

To explore the robustness of the results regarding the primary outcome, we conducted sensitivity analyses in the (a) per-protocol sample and subgroups of participants, (b) who fulfilled the criteria of at least one psychological disorder at baseline, or (c) who were in concurrent psychotherapy at baseline. Observed and estimated means for the primary outcome assessed at baseline and post-assessment and effect sizes for within- and between-group differences are displayed in Supplementary Table S23. Furthermore, the mixed effects models are summarized in Supplementary Tables S25–S27. The same result pattern emerged for all three subgroups regarding the primary outcome when comparing the intervention groups with the control group. When comparing both intervention groups with each other, scenarios A and C revealed differences between GU and AM equivalent to the primary analyses. However, in scenario B, the difference between GU and AM at post-assessment was only borderline significant, (*t*(99) = 1.90, *p* = 0.06, *d* = 0.50) (see Supplementary Table S24 for a summary of the contrast analyses).

### Reliable improvement and deterioration

Reliable improvement (pre-post change UCLA-9 > 2.93) in the ITT-sample (*n* = 243), did not significantly differ between GU (47/98, 48.0%), AM (40/97, 41.2%), and WL (15/48, 31.3%; *χ*^2^(2) = 3.73, *p* = 0.15, *V* = 0.12). In terms of deterioration, a significant difference between GU (2/98, 2.0%), AM (5/97, 5.2%) and WL (6/48, 12.5%; *χ*^2^(2) = 6.97, *p* = 0.03, *V* = 0.17) was observed. Participants in the GU condition had a significantly lower probability of deterioration in loneliness from pre to post compared to the WL condition (*p* = 0.02, OR 0.15, 95% CI 0.02–0.73).

In the per-protocol sample (*n* = 172, i.e., participants who completed both baseline and post-assessment and accessed at least four modules) reliable improvement significantly differed between the three conditions (GU: 42/69, 60.9%; AM: 30/57, 52.6%; WL: 14/46, 30.4%; *χ*^2^(2) = 10.46, *p* = 0.005, *V* = 0.25). Participants in the GU (*p* = 0.002, OR 3.50, 95%-CI 1.60–7.96) and in the AM condition (*p* = 0.03, OR = 2.51, 95%-CI 1.12–5.82) had significantly higher probabilities for reliable improvement regarding loneliness than the participants in the WL condition. Concerning deterioration, a significant difference between conditions was observed (GU: 1/69, 1.4%; AM: 4/57, 7.0%; WL: 6/46, 13.4%; Fisher’s Exact Test: *p* = 0.04). The probability for deterioration was significantly lower for participants in the GU (*p* = 0.02, OR 0.11, 95% CI 0.00–0.71) compared to the WL condition.

### Participant satisfaction and negative effects

Regarding the satisfaction with the program (CSQ-8) assessed at post, participants in the GU (*n* = 65, *M* = 3.18, *SD* = 0.61) and AM condition (*n* = 60, *M* = 3.02, *SD* = 0.55) indicated to be generally satisfied with the treatment they received. The two groups did not significantly differ regarding their satisfaction with the program (*t*(123) = − 1.59, *p* = 0.12). Both intervention groups rated the usability of the program as “good”^39^, and there were no significant differences between the conditions (GU: *n* = 66, *M* = 80.17, *SD* = 15.68; AM: *n* = 60, *M* = 79.44, *SD* = 16.02; *t*(124) = − 0.26, *p* = 0.80).

Due to a programming error, data regarding negative effects of eight participants in the GU and four participants in the AM condition could not be included in the analyses. Negative effects at post-assessment were computed for the completer sample. At post-assessment, participants in the GU (*n* = 62, *M* = 0.32, *SD* = 0.81) and AM condition (*n* = 60, *M* = 0.35, *SD* = 0.88) did not significantly differ concerning the mean number of reported negative effects due to the program (*t*(120) = 0.18, *p* = 0.86). A total of 13 (21.0%) participants in the GU and 15 (25.0%) in the AM condition did report at least one negative effect that they attributed to the self-help program. The number of negative effects reported ranged from 0 to 5 in the GU and 0–6 in the AM condition. Most frequently, participants (GU: *n* = 9, 14.5%, AM: *n* = 6, 10.0%) reported having experienced prolonged periods during the intervention phase when they felt bad (item 13). The second most mentioned was that they would suffer more from events from the past (GU: *n* = 5, 8.1%, AM: *n* = 3, 5.0%).

## Discussion

The current study evaluated the effects of a 10-week Internet-based self-help intervention with human guidance or automated messages compared to a waitlist control group for people suffering from loneliness. At post-assessment, the pooled intervention groups showed significantly reduced loneliness compared to the control group. This finding was robust across several sensitivity analyses. Moreover, this study shows the superiority of human guidance versus automated messages in ICBT against loneliness for the first time. Regarding the secondary outcomes, the intervention groups showed reduced depressive symptoms, social anxiety symptoms, social avoidance behavior, and rejection sensitivity at post-assessment compared to the waitlist control group. However, no significant differences in secondary outcomes at post-assessment were observed between the intervention groups. Satisfaction with the intervention was generally high and usability was rated as good in both intervention conditions, but no significant differences between intervention conditions were observed.

The greater decrease in loneliness in both intervention groups was according to our hypothesis, and further supports initial findings from Swedish trials on the efficacy of ICBT for reducing loneliness. The Swedish trials found moderate effects sizes (*d* = 0.77^29^ and *d* = 0.71^30^) in favor of the guided ICBT compared to the waitlist control group, which is comparable to the current study with a moderate between-group effect size for the pooled intervention versus WL condition (*d* = 0.57, 95% CI [0.25; 0.89]) and a large effect size when comparing the guided ICBT with the WL (*d* = − 0.80, 95% CI [− 1.16; − 0.44]). Thus, the results of the current study indicate that loneliness can be reduced with ICBT.

As hypothesized, the guided condition was superior to the automated message condition (*d* = − 0.42, 95% CI [− 0.70; − 0.13]). This finding provides evidence for the first time on the role of human contact (i.e., guidance) in loneliness reduction with ICBT. It is possible that participants in the guided condition experienced aspects relevant to satisfying social relationships (e.g., being valued and understood by the coaches), which directly led to a more substantial reduction in loneliness. However, they might also have felt accountable to the coach and thus used the self-help program more intensively and thoroughly, leading indirectly to a reduction in loneliness. The latter might be reflected in the time spent within the program, which was significantly higher in the guided than in the automated message condition. This aligns with previous studies highlighting increased adherence in guided versus automated message/unguided conditions for various mental health interventions^34^. A recent study in people suffering from depressive symptom further showed that both therapeutic alliance and adherence mediated the effect of guidance^40^. However, in the aforementioned studies, adherence was operationalized as the completion rate of modules^34^ or as a composite score consisting of number of clicks, number of topics worked on, number of completed exercises, and time spent within the program^40^, respectively. The number of modules accessed yielded non-significant differences between study groups in the current study. This points to the relevance of carefully considering the operationalization of adherence and adds to the ongoing discussion on how to conceptualize best and assess adherence in internet-based interventions^41^. Overall, investigating the direct and indirect effects of guidance on the reduction of loneliness merits further investigation.

Loneliness was assessed by different means in the current trial. An indirect measure of loneliness (i.e., without mentioning “lonely”) was administered as the primary outcome. Furthermore, we directly asked the participants how often they felt lonely. While a significant difference at post-assessment on the indirect measure of loneliness was found between the intervention and waitlist control conditions, no difference between groups was observed in the direct assessment of loneliness. The significant reduction in the direct measure of loneliness within all three study conditions may have contributed to the absence of group differences at post. Accordingly, the choice of measure for loneliness could be of relevance in intervention research. Prior research^42^ already highlighted diverging results, e.g., regarding the prevalence of loneliness if measured directly or indirectly. Therefore, a better understanding of the constructs captured with indirect and direct measures of loneliness is needed.

Concerning secondary outcomes, the intervention groups showed significantly reduced depressive symptoms at post-assessment compared to the control group. A decrease in depressive symptoms has also been reported for the ICBT condition compared to the control group in the study by Käll and colleagues^30^. Furthermore, in line with another trial^29^, we found a significant reduction in social anxiety symptoms in the intervention groups compared to the waitlist. Findings of previous observational studies^11,12,43^ point to the interrelatedness between loneliness, depression, and social anxiety. Decreasing loneliness might thus relate to changes in social anxiety and depression. However, further research is needed to clarify whether the intervention for loneliness directly affects symptoms of anxiety and depression or if changes in loneliness influence those symptoms. This would shed light on the relationship between loneliness and social anxiety and depression and, furthermore, allow to improve interventions for lonely individuals with comorbid social anxiety or depression.

Regarding further secondary outcomes, the intervention groups showed reduced social avoidance behavior and rejection sensitivity at post-assessment compared to the waitlist control group. Concerning other secondary outcomes associated with the cognitive model of loneliness (e.g., interpretation bias), comparing the intervention conditions to the control group and both intervention groups with each other did not show significant differences at post-assessment. Since loneliness is a complex phenomenon, it is possible that changes in secondary outcomes have taken place at the individual level but are not reflected at the group level. Possible sources of the complexity of loneliness are the various causes that can lead to loneliness, e.g., the death of a close relative, a small social network, or feelings of personal inadequacy^44^. Depending on the underlying causes and individual circumstances, taking different approaches to break the vicious cycle of loneliness might be necessary^45^. A person with a small social network may need different strategies to reduce their loneliness than someone with a larger social network. A better understanding of the causes, circumstances, and characteristics of lonely individuals would allow tailoring interventions to the needs of those individuals.

It is also noteworthy that loneliness was reduced in the intervention groups, although there was no change in the size of the social network at the mean level. This supports the current literature indicating that the attitude towards oneself and the quality of social relationships may be more relevant to feelings of loneliness than the number of social relationships. Thus, interventions aiming solely at increasing social contact might not reduce feelings of loneliness^23^. Furthermore, despite the significant mean change in loneliness in favor of the intervention groups, it also became evident that some participants profited more from the respective intervention than others. Almost half of the participants in the guided and about 40% in the automated messages condition showed a reliable improvement at post-assessment. Accordingly, the object of future research should be to identify predictors and moderators of a reduction in loneliness. This would help to better understand for whom ICBT to alleviate loneliness would be suitable or who would benefit from additional human guidance.

The results of the present study should be considered in the light of several limitations. First, we excluded participants presenting severe depressive symptoms at baseline, preventing us from generalizing our results to lonely individuals with severe depressive symptoms. Second, we investigated a self-selected sample, which might imply that only individuals highly motivated to use an Internet-based self-help intervention participated in the study. Our results thus mainly apply to individuals motivated to work on their feelings of loneliness using an Internet-based self-help intervention. Third, our sample was predominantly female and highly educated. Thus, it did not represent the entire bandwidth of individuals experiencing loneliness. However, almost 70% of the sample showed higher baseline loneliness scores than 95% of the German general population, implying that we recruited a highly burdened subsample of lonely individuals. Fourth, the present results only allow us to conclude the short-term effects of the intervention. However, participants in both intervention conditions completed questionnaires 6 and 12 months after randomization, and the results of follow-up assessments will be disseminated later. Finally, since self-report measures were used for data collection, it cannot be ruled out that people may have given socially desirable answers, and thus the data may have been biased. However, since loneliness is a purely subjective feeling, self-report measures on loneliness are essential.

In conclusion, the present study demonstrated that an Internet-based self-help intervention mainly based on CBT principles effectively reduces loneliness. The findings add to the existing evidence on the efficacy of ICBT in reducing loneliness, and they advance existing knowledge by showing that compared to automated messages, human support is associated with lower loneliness scores after the intervention. Since the Internet-based self-help intervention not only reduced loneliness but also decreased depressive and social anxiety symptoms, alleviating loneliness using ICBT might thus also contribute to the reduction of the overall burden of mental health disorders.

## Methods

### Study design

**Figure 2:**
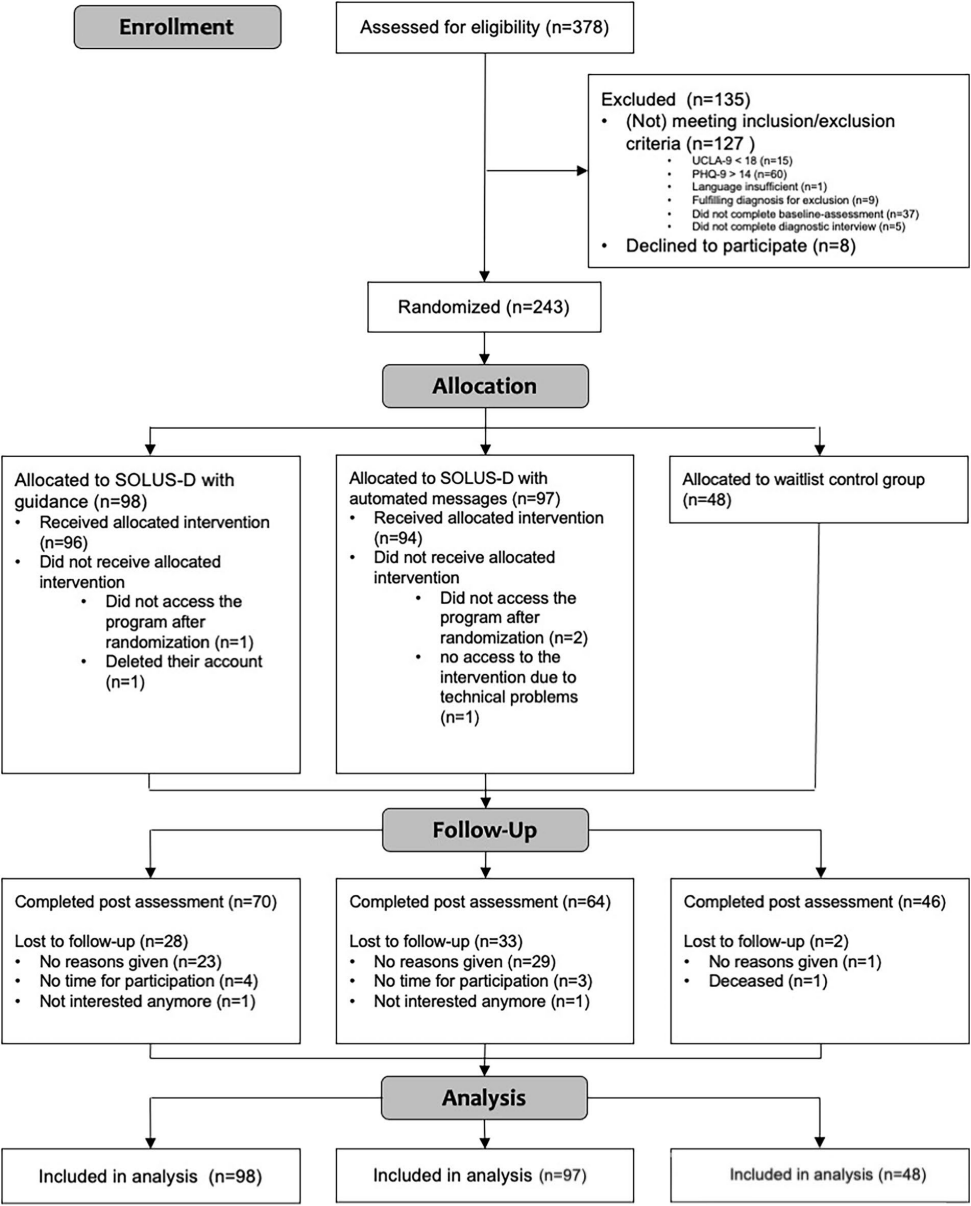
CONSORT (Consolidated Standards of Reporting Trials) diagram. Post = week 10 assessment.

We conducted a 10-week randomized controlled trial (RCT) using a parallel-group design, comparing two active intervention conditions to a waitlist control condition (see Fig. 2). Both intervention conditions had immediate access to the self-help program, and participants in the waitlist control group were given full access 10 weeks after randomization.

This trial was conducted and reported following the CONSORT-SPI 2018 checklist^46^. This trial was preregistered with clinicaltrials.gov (NCT04655196, registration date: 07/12/2020), conducted in accordance with the declaration of Helsinki, and has been approved by the Cantonal Ethics Committee Bern (CEC; ID: 202-01298). Moreover, we published a study protocol^47^. Informed consent was obtained from all subjects before participating in the study.

### Participants and procedure

To be included in the study, individuals had to be at least 18 years old, score 18 or higher on the UCLA Loneliness Scale-9 item version (UCLA-9), have sufficient knowledge of German, have access to the Internet and an Internet-enabled device, and provide a signed consent form and a contact person in case of emergency. Individuals with current severe depressive symptoms, a lifetime diagnosis of psychotic or bipolar disorder, fulfilling the criteria for a current severe substance use disorder, or reporting acute suicidal plans were excluded from the study. Depressive symptoms were assessed with the PHQ-9, and the other exclusion criteria were evaluated with the diagnostic interview (Mini-DIPS-Open Access^48^). All study participants were allowed to use additional therapeutic services and medication.

Between May 17, 2021, and July 31, 2022, we recruited 243 participants from the general population in German-speaking countries. Participants were recruited via social media, articles/interviews in newspapers, radio interviews, newsletters, google-ads, the study website, and the website listing ongoing studies from our research hub. After registering on the study website and returning a signed informed consent via email or post, interested participants received an email link to the baseline assessment. Trained and supervised master- and doctoral students conducted diagnostic interviews (Mini-DIPS-Open Access^48^) via telephone with all participants who completed the baseline assessment to assess diagnoses relevant to exclusion from the study. After the diagnostic interview, eligible participants were automatically block-wise randomized with Qualtrics^49^ to either the two intervention conditions (Internet-based self-help program with human guidance or automated messages) or the waitlist control group. After the group allocation, participants in the intervention conditions had access to all modules of the Internet-based self-help program. In addition to the baseline assessment, all participants were asked to complete further assessments at 10 weeks (post) after the randomization. After completing the post-assessment, the waitlist control group received access to the intervention in a self-guided format. After randomization, participants and coaches delivering guidance were not blinded concerning the corresponding group allocation. Participants were not compensated for partaking in the trial. There was no face-to-face contact between participants and the study team throughout the study period. All communication took place via email, telephone, or via the message function within the self-help platform.

Out of 378 potential participants, 243 met all inclusion and no exclusion criteria and were eligible to participate. In total, 98 participants were randomly assigned to the guidance condition, 97 to the automated message condition, and 48 to the waitlist condition (see Fig. 2). Socio-demographics are reported in Table 1. The sample was mainly female (*n* = 191, 78.6%), living alone (*n* = 153, 63.0%), single (*n* = 182, 74.9%), and had a university degree (*n* = 151, 62.4%). Participants were between 19 and 80, with a mean age of 45.77 (*SD* = 14.85) years. A total of 79 (32.5%) were in psychological treatment at baseline, and 125 (51.4%) fulfilled the criteria of at least one psychological disorder according to the diagnostic interview (Mini-DIPS OA). The most prevalent was social anxiety disorder (*n* = 71, 29.2%). On average, participants experienced loneliness for 11.62 years (*n* = 238, *SD* = 13.91, *Md* = 5.25).

### Intervention—SOLUS-D

SOLUS-D. The Internet-based self-help program SOLUS-D is a German adapted version of an Internet-based self-help program developed and pilot-tested in Sweden^29^. The program content is mainly based on cognitive behavioral principles. Compared to the original version, SOLUS-D contains additional modules focusing on mindfulness, self-compassion, and social skills relevant to building or deepening social relationships. SOLUS-D consists of nine modules that are mainly text-based and contain video and audio elements. Each module delivers theoretical information with a specific thematic focus, whereby this content can be deepened and transferred to everyday life with practical exercises. An integrated diary function was additionally aimed at changing the attentional focus and becoming more aware of compassion for the self and others in everyday life. A detailed description of the program content can be found in Supplementary Table S1. We recommended working on one module per week, corresponding to an approximate weekly time commitment of 50 min. However, participants could spend more time on the modules, corresponding exercises, and diaries. As the modules build on each other, we recommended working on them in a specific sequence. However, as the order of the program content might not suit everyone, all modules were unlocked from the beginning rather than every week. Participants were free to repeat content and exercises upon their preferences. The program was accessible by computer, smartphone, or tablet. Secure Socket Layer encryption was used to secure Internet-based communication with the program and the guides. Within the program, participants were only identifiable with anonymous login names, and they had a personal, password-protected login for the program.

### Study conditions

Participants in the “*Guidance*”- condition (GU) had access to SOLUS-D 1 day after randomization. They received weekly individualized feedback (i.e., guidance) from trained and supervised coaches through the message function of the self-help program. Participants were informed via the study information and after group allocation that a coach sent the weekly messages. The messages entailed feedback on participants' work within the program during the previous week and answered individual questions. An example of a weekly guidance message can be found in the online Supplementary Material. The primary aim of the guidance was to motivate participants to continue with the program. The main content of the messages was semi-structured and manualized according to the theoretical model of Supportive Accountability^50^. This model aims to increase adherence through human contact by being accountable to a coach. The coaches sent participants who did not log into the program in the previous week a standardized reminder. Reminders were sent for up to three consecutive weeks if participants did not log into the program or react to the reminders. The coaches were two psychologists with a master’s degree in their first year of a CBT post-graduate program and ten master’s students in their last term of a graduate program in clinical psychology. The authors NS, AS, and TK trained and supervised the coaches. On average, the coaches spent 17.10 min (*SD* = 10.15, *Md* = 14.25) on guidance per participant per week.

Participants in the “*Automated Message*”-condition (AM) had access to SOLUS-D 1 day after randomization and received weekly standardized messages via email. Participants were informed via the study information and after group allocation that the weekly messages were sent automatically and not by a study team member, i.e., a human being. The automated messages aimed to motivate participants to continue working with the program, e.g., by summarizing the module contents of the previous week and providing an outlook of the next module. An example of a weekly automated message can be found in the online Supplementary Material. After receiving access to the intervention, participants in the AM conditions were informed that upcoming technical problems could be addressed to the study team.

Participants in the “*Waitlist Control Group*” (WL) received access to the intervention in a unguided format, i.e., without guidance or automated messages, 10 weeks after randomization upon completing the post-assessment. After receiving access to the intervention, participants in the WL condition were informed that upcoming technical problems or questions regarding the program could be addressed to the study team.

### Measures

Demographic variables (e.g., gender, age, and education level) and therapy and medication status were self-reported by the participants at baseline. Self-reported primary and secondary outcome measures were assessed at baseline, and 10 weeks after randomization. Participants who did not respond to the assessment invitation received up to three weekly reminders via email. All questionnaires were administered in German and completed on the online survey platform Qualtrics^49^ by the participants. The diagnostic interview was administered via telephone.

#### Primary outcome

*Loneliness*, measured at the post-assessment timepoint, was the primary outcome and was assessed with the 9-item short version (UCLA-9)^51^ of the UCLA Loneliness Scale^52,53^. The original scale consists of 20 Items and assesses three dimensions of loneliness: intimate, relational, and collective. The nine-item version consists of the three items with the highest factor loadings on each facet of loneliness^54^. The validity and reliability of the short version are comparable with those of the 20-item original scale^55^. The response options are (1) never, (2) rarely, (3) sometimes, and (4) always. After recoding reverse-coded items, all items are summed up, and the total score ranges from 9 to 36, with higher values indicating more pronounced feelings of loneliness. Cronbach’s α for the UCLA-9 at post-assessment was 0.83. Internal consistency at post-assessment is reported since baseline data were affected by range restriction and biased reliability since we used the UCLA-9 as an inclusion criterion^56^. As previous studies detected differences, e.g., in the prevalence of loneliness, depending on either directly (i.e., using the word “lonely”) or indirectly (i.e., not mentioning the word “lonely”) measuring loneliness^42^, an additional single item was administered to assess *loneliness directly*. Furthermore, an additional 3-item short form of the UCLA Loneliness Scale (UCLA-3)^57^ was used, as norms for the German population exist^38^.

#### Secondary outcomes

*Depressive symptoms* were assessed with the 9-item depression module of the Patient Health Questionnaire (PHQ-9)^58,59^. The short form of the Social Interaction Anxiety and Social Phobia Scale (SIAS-6 & SPS-6)^60^ was used to assess symptoms of *social anxiety*. *Satisfaction with life* was measured with the 5-item Satisfaction with Life Scale (SWLS)^61,62^. Furthermore, we assessed *self-esteem* with the 10-item revised German version^63^ of the Rosenberg Self-Esteem Scale (RSES)^64^ and used the 20-item Sussex-Oxford Compassion for the Self Scale (SOCS-S)^65^ to measure *self-compassion*. The Social Network Index (SNI)^66^ was administered to assess *objective social isolation*, i.e., network size^67^. We used the Personality Inventory for the DSM-5 Brief Form Plus (PID5BF+)^68^ to assess *maladaptive personality traits*. *Interpretation bias* was assessed with the respective subscale of the Interpretation and Judgmental Bias Questionnaire (IJQ)^69,70^. The Adult-Rejection Sensitivity Questionnaire (A-RSQ)^71^ was used to measure *rejection sensitivity*. Furthermore, we administered the subscale Behavior-social avoidance of the Cognitive-Behavioral Avoidance Scale (CBAS)^72,73^ to assess *social avoidance behavior.* The Distress Disclosure Index (DDI)^74^ measured *comfort with self-disclosure*. We administered the Kernis Goldman Authenticity Inventory-short form (KGAI-SF)^75^ to assess *authenticity*. We used the corresponding subscale of the Bern Embitterment Inventory (BVI)^76^ to assess *misanthropy*. *Self-determined motivation for solitude* was assessed with the respective subscale from the Motivation for Solitude Scale-Short Form (MSS-SF)^77^.

#### Further measures

At post-assessment, participants in both intervention groups completed measures on *client satisfaction* (CSQ-8)^78^ and *usability* (SUS)^39^ of the intervention. Moreover, we assessed *negative effects* that occurred during the intervention phase and were attributed to the intervention by participants in the intervention conditions with the INEP^79^ at post-assessment. *Adherence* to the Internet-based program was assessed as the number of modules completed. A module was considered completed when each page per module had been clicked at least once. Furthermore, the time participants spent within the program was measured. The coaches noted down the amount of time they spent reading the participants’ content within the program, as well as writing and delivering guidance. Before randomization, we administered the Mini-DIPS-Open Access^48^ to assess *diagnoses of mental disorders.* We refer to the online Supplementary Material and the study protocol^47^ for a more detailed description of all measures.

### Statistical analyses

Following the intention-to-treat principle (ITT), we included all randomized participants in the primary analyses. We computed ANOVAs for continuous and Chi-Square tests for nominal data to assess group differences at baseline and group comparisons regarding reliable change. Independent sample t-tests were performed to determine group differences in program usage, satisfaction with the program, and negative effects due to the intervention. Where relevant assumptions for the respective tests were violated, we conducted non-parametric tests, e.g., Fisher’s Exact Test or Kruskal–Wallis Test. For the primary analyses, we used linear mixed models with restricted information maximum likelihood estimation in the *lme4* package^80^ in R (version 4.2.1) to evaluate change in the primary and secondary outcome variables. Linear mixed models are suitable for longitudinal data with repeated measures, as the dependency of the data is accounted for^81^. Furthermore, linear mixed models yield robust estimates despite missing data, accounting for it through maximum likelihood estimation, which produces unbiased estimates under the Missing at Random (MAR) assumption^82^. We estimated linear mixed models for the primary and each secondary outcome separately with fixed effects of time, condition, Time × Group interaction, and random intercepts for participants to evaluate the efficacy of the intervention. Time and condition were entered into the models as categorical variables. We did not include random slopes as the convergence of the model could not be achieved. Significant Time × Group interactions were followed up with planned contrast analyses, where we compared the two intervention conditions against the waitlist condition (GU: − 0.5, AM = − 0.5, WL = 1) and the two intervention conditions against each other (GU: − 1, AM = 1, WL = 0). Following Feingold^83^, between-group effect sizes (i.e., Cohen’s *d*) were calculated by dividing the estimated mean difference at post-assessment by the pooled standard deviation at baseline. Within-group effect sizes were calculated by dividing the difference between the estimated means (pre-post) by the pooled standard deviation of the observed means from both time points. Additionally, we estimated 95% confidence intervals for the effect sizes. The α error level was set to 0.05. Only the primary outcome measure and the PHQ-9 required participants to answer all items. This was not the case for the other questionnaires to reduce the attrition rate. Accordingly, for scales with missing values at the item level, the scale scores were calculated with the available data^84^.

Reliable improvement or deterioration in the primary outcome was calculated using the reliable change index (RCI)^85^. To determine the reliable change index, we used Cronbach’s alpha (0.90) of the UCLA-9 from a sample of the general population of German-speaking countries (*n* = 813, unpublished data) and the current study samples' standard deviation at baseline (*SD* = 3.34). Participants with change scores (pre-post) greater than 2.93 on the UCLA-9 were classified as reliably improved, not changed when scoring between 2.93 and − 2.93, and deteriorated with a change score lower than − 2.93. To ensure a conservative estimate of the change in loneliness, reliable change was computed using the ITT sample, replacing missing values at post-assessment with the last observation carried forward. Additionally, reliable change was calculated in the per-protocol sample consisting of participants who completed the baseline- and post-assessment and logged into four or more modules (i.e., minimal therapeutic contact).

High dropout rates in studies on Internet-based self-help programs are common and can lead to biased results. To check the robustness of the results, we additionally conducted sensitivity analyses and ran the primary analyses with the per-protocol sample. We conducted further sensitivity analyses focusing on different subgroups, i.e., participants fulfilling at least one psychological disorder and participants indicating to attend psychotherapeutic treatment at baseline.

### Sample size and power

We conducted an a priori power analysis using G*Power 3^86^ and aimed at detecting small effect sizes^87^ of *f* = 0.10 (equivalent to Cohen *d* = 0.20) for the Time × Group interaction for the two intervention conditions at an α error level of 0.05., a power (1 − *β*) of 0.80, and with correlations of *r* = 0.60 between pre-and post-treatment measures, as found in a previously conducted trial on ICBT for loneliness^29^. According to the power analysis, a sample size of 80 participants per intervention group was sufficient to detect statistically significant differences with these assumptions. Furthermore, to account for dropouts of approximately 25%, we decided to randomize 100 participants to each intervention group. Concerning the comparison between the intervention and waitlist control groups, 50 participants were considered sufficient for the waitlist since between-group effects were expected to be medium-to-large, based on the Swedish trials mentioned above^29,30^. Thus, we intended to randomize 250 participants (randomization ratio: 2:2:1). For regulatory reasons, we had to end recruitment when 243 participants were randomized, which might have limited our ability to detect the intended effects.

### Supplementary Information

Supplementary Information.

## Data Availability

De-identified data and statistical codes supporting the findings of this study will be made available upon publication of the manuscript on OSF (https://osf.io/tmk5e/). Information on demographics and diagnoses is not included in this data set to ensure the privacy of participants.
