# Supplementary material for: Efficacy of an Internet-based self-help intervention with human guidance or automated messages to alleviate loneliness: a three-armed randomized controlled trial
Source: Sci Rep. 2024 Mar 19;14:6569. doi: 10.1038/s41598-024-57254-0 (PMC10951227; doi:10.1038/s41598-024-57254-0)
Supplement: Supplementary file 1 — Supplementary Information. [file 41598_2024_57254_MOESM1_ESM.docx]

Supplementary Materials

Efficacy of an Internet-based Self-help Intervention with Human Guidance or Automated Messages to Alleviate Loneliness: A Three-Armed Randomized Controlled Trial

Noëmi Seewer*, Andrej Skoko, Anton Käll, Gerhard Andersson, Maike Luhmann, Thomas Berger, and Tobias Krieger

*Corresponding author; noemi.seewer@unibe.ch

Table of Content

[Methods 3](#_Toc157425613)

[Intervention – SOLUS-D 3](#_Toc157425614)

[Table S1 3](#_Toc157425615)

[Study conditions 5](#_Toc157425616)

[Example of a weekly feedback in the guided condition 5](#_Toc157425617)

[Example of a weekly feedback in the automated message condition 5](#_Toc157425618)

[Measures 6](#_Toc157425619)

[Results 10](#_Toc157425620)

[Baseline Evaluation and Preliminary Analyses 10](#_Toc157425621)

[Table S2 10](#_Toc157425622)

[Study Dropout Analysis 11](#_Toc157425623)

[Table S3 11](#_Toc157425624)

[Table S4 12](#_Toc157425625)

[Mixed effects models 14](#_Toc157425626)

[Table S5 14](#_Toc157425627)

[Table S6 14](#_Toc157425628)

[Table S7 15](#_Toc157425629)

[Table S8 16](#_Toc157425630)

[Table S9 16](#_Toc157425631)

[Table S10 17](#_Toc157425632)

[Table S11 17](#_Toc157425633)

[Table S12 18](#_Toc157425634)

[Table S13 19](#_Toc157425635)

[Table S14 20](#_Toc157425636)

[Table S15 20](#_Toc157425637)

[Table S16 21](#_Toc157425638)

[Table S17 21](#_Toc157425639)

[Table S18 22](#_Toc157425640)

[Table S19 23](#_Toc157425641)

[Table S20 23](#_Toc157425642)

[Table S21 24](#_Toc157425643)

[Contrast Analyses Primary and Secondary Outcomes 26](#_Toc157425644)

[Table S22 26](#_Toc157425645)

[Sensitivity Analyses 27](#_Toc157425646)

[Table S23 27](#_Toc157425647)

[Table S24 28](#_Toc157425648)

[Table S25 29](#_Toc157425649)

[Table S26 29](#_Toc157425650)

[Table S27 30](#_Toc157425651)

[References 32](#_Toc157425652)

# Methods

## Intervention – SOLUS-D

### Table S1

*Content of SOLUS-D* .

| Modul | Title | Content | Exercises |
| --- | --- | --- | --- |
| 1 | Loneliness and personal values | This module includes information on how to use the program, the structure of the intervention, psychoeducation on loneliness, and personal values. | - Vicious cycle of loneliness - Values in different life areas - Introduction Mood-Diary (continuous exercise) |
| 2 | Goal setting and mindfulness | Personal goals are set, and a theoretical and practical introduction to mindfulness is provided. | - Setting goals - Mindful Breathing - Body-Scan - 3 Minute Breathing Space |
| 3 | Self-compassion | Self-compassion is introduced theoretically and practically. | - Kindness Meditation: Self-compassion - LKM - Introduction (self-) compassion diary (continuous exercise) |
| 4 | Acceptance of loneliness and solitude | The importance of emotions is emphasized and a strategy for accepting emotions is introduced. There is also a reflection on time spent alone. | - Accepting Emotions - Exposition with time spent alone - Reframing time spent alone |
| 5 | Identifying and changing thoughts | The effects of negative automatic thoughts and the relationship between thoughts, experiences, and behavior are introduced. Dysfunctional thoughts are identified and restructured. | - Identifying NAT - Challenging NAT and formulating alternative thoughts |
| 6 | Rumination and behavioral experiments | Strategies for dealing with rumination and the rationale of behavioral experiments are introduced. | - Disrupting rumination - Behavioral experiments |
| 7 | Social relationships and feeling connected | The current social relationship situation is evaluated, values in social relationships are identified, and different social relationship skills relevant to building and maintaining valued social relationships are introduced. | - Social convoy - Values in close relationships - Boundaries |
| 8 | Building social activities | The relationship between behavior and loneliness is explored, avoidance behavior and passivity are addressed, and value-based social activities are introduced. | - Avoidance and passivity - Values based behavioral activation |
| 9 | More social activities and further goals | Obstacles to behavioral activation are addressed and new activities can be planned. The contents of the past modules are summarized and goals for further engagement with loneliness can be set. | - Values based behavioral activation - Formulating further goals - Strategy tool box |
| Note: This table is adapted from a table in a previous publication: Seewer et al., ^[1]^ which is published under Creative Commons Attribution 4.0 International License ^[2]^. | | | |

*Note.* LKM = Loving Kindness Meditation, NAT = negative automatic thought.

## Study conditions

### Example of a weekly feedback in the guided condition

*Hello @user-name*

*I have seen that you have been working very intensively with the program this week. I am very pleased about that. You have examined your automatic negative thoughts in detail, observed them in everyday life and tried to challenge them. You have done this really well. I noticed that you left the field for alternative thoughts empty and wondered whether you found this part of the exercise challenging. This is quite normal and takes practice to be able to formulate a thought that feels appropriate. If you keep trying to observe your thoughts, it will become easier to challenge them and formulate alternative thoughts over time. You are welcome to continue with the next module; in module 6, we will deal with ruminative thoughts and introduce you to another strategy for challenging automatic thoughts. If you have any questions or comments, please do not hesitate to contact me.*

*I wish you all the best for your work with Module 6.*

*Best regards*

*Name of the coach*

*SOLUS study team*

### Example of a weekly feedback in the automated message condition

*Hello @user_name*

*Congratulations! You have probably already completed the fifth module and are halfway through the program. You may have found some content exciting and others less relevant to your current situation. This is quite normal, as loneliness manifests itself very differently in different people and has different causes, and therefore, not all those affected will find the same things helpful. Nevertheless, continue to use the program. In Module 5, we looked at our thoughts. This is important because our interpretations of situations influence how we behave and feel. Recognising automatic negative thoughts can be very challenging at the beginning precisely because these thoughts are so automatic and sometimes barely conscious. This is precisely where mindfulness practice can be supportive, as we can use it to sharpen our awareness of our thoughts. Formulating alternative, more helpful thoughts often doesn't work right from the start and is also a matter of practice. Be kind to yourself, and just keep trying. We hope that practising will pay off. You are now welcome to continue with Module 6. In it, we will learn about typical thought traps we often encounter when challenging our thoughts. We also encourage you to conduct behavioural experiments to check automatic thoughts.*

*We wish you an interesting week!*

*Best regards*

*Your SOLUS study team*

*This is an automated message. Please do not reply to this message. However, please do not hesitate to contact the SOLUS study team in case of technical problems or uncertainties.*

## Measures

***Patient Health Questionnaire (PHQ-9):*** The PHQ-9 is a 9-item self-report measure of depressive symptoms ^[3,4]^. Each item represents one of the nine Diagnostic and Statistical Manual of Mental Disorders (DSM)-IV criteria for depression. The sum scores of the scale range from 0-27, with higher scores indicating more severe depressive symptomatology. The PHQ-9 shows good validity ^[3]^ and is sensitive to change ^[4]^. Cronbach’s *α* for the PHQ-9 at post-assessment was 0.76.

***Social Interaction Anxiety and Social Phobia Scale – Short from (SIAS-6/SPS-6):*** The short form of the Social Interaction Anxiety and Social Phobia Scale ^[5]^ assesses symptoms of social anxiety. The two scales are often administered together and complement each other. The 12 items are rated on a 5-point Likert scale with sum scores ranging from 0 to 48. Higher scores indicative more pronounced symptoms of social anxiety. The scales show good psychometric properties and are sensitive to change over time ^[5]^. Cronbach’s *α* for the SIAS-6 at post-assessment was 0.78 and 0.83 for the SPS-6 respectively.

***Satisfaction with Life Scale (SWLS):*** The SWLS is a 5-item scale to assess satisfaction with life ^[6,7]^. Items are rated on a 7-point Likert scale ranging from 1 (strongly disagree) to 7 (strongly agree). Items are summed up and higher scores indicate greater satisfaction with life. This scale has good psychometric properties and norm values based on a large German sample ^[8]^. Cronbach’s *α* for the SWLS at post-assessment was 0.87.

***Rosenberg Self-Esteem Scale (RSES):*** We used the 10-item revised German version ^[9]^ of the RSES ^[10]^ to assess global self-esteem. The scale measures positive and negative aspects of self-esteem. The items are rated on a 4-point Likert scale ranging from 0 (strongly disagree) to 3 (strongly agree). Higher mean scores indicate greater self-esteem. Cronbach’s *α* for the RSES at post-assessment was 0.91.

***Sussex-Oxford Compassion for the Self Scale (SOCS-S):*** The SOCS is a 20-item scale to measure self-compassion for the self ^[11]^. Items are rated on a 5-point Likert scale with response options ranging form 1 (not at all true) to 5 (always true). The scale allows to measure five dimensions of self-compassion: (1) recognizing suffering, (2) understanding the universality of suffering, (3) feelings for the person in suffering, (4) tolerating uncomfortable feelings, and (5) motivation to act to alleviate suffering. The SOCS is valid and shows adequate psychometric properties ^[11]^. Cronbach’s *α* for the SOCS-S at post-assessment was 0.91.

***Social Network Index (SNI):*** The SNI ^[12]^ is a 12-item scale to assess different types of social relationships (e.g., partner, parents, parents-in-law, children, other close relatives, close neighbors, and friends). First, respondents indicate how many relationships of each type they have, and second, indicate how many of these people they have contact with at least once every two weeks. The questions are answered on a scale ranging from “0” to “7 or more”, apart from parents and in-laws, who are restricted to two, and for the items on romantic relationship, where only a yes or no answer is allowed. Three subscales can be computed: network size, network diversity, and number of embedded networks. Network size is a proxy for social isolation and is determined by the number of people with whom the person has contact at least once every two weeks. The number of social roles of a person is described by network diversity. For this purpose, the number of areas in which the person maintains regular contacts with at least one person is added up. The number of embedded networks reflects the number of different network areas in which the person is active. Activity in the different areas is defined by having at least four high contact persons within each area. Higher scores indicate larger network size, diversity, or high number of embedded networks ^[13]^.

**Personality Inventory for the DSM-5 Brief Form Plus (PID5BF+)**: The PID5BF+ ^[14]^ is a 34-item short version of the Personality Inventory for DSM-5 ^[15]^. The short form assesses five domains and 15 facets in accordance with criterion B of the Alternative Model of Personality Disorders included in the DSM-5. The respective domains are Negative Affectivity, Detachment, Antagonism, Disinhibition, and Psychoticism. Additionally, the domain Anankastia of the International Classification of Diseases (ICD)-11 is included. All items are rated on a 4-point Likert scale with responses ranging from 0 (very false or often false) to 3 (very true or often true). In the current study, the average score of the 6 domains was used to indicate the severity of personality dysfunction according to the DSM-5 section III and the ICD-11 classification of Personality Disorders ^[16]^. Cronbach’s *α* for the PID5BF+ at post-assessment was 0.86.

**Interpretation and Judgmental Bias Questionnaire (IJQ)**: The IJQ ^[17,18]^ is a 24-item scale measuring interpretation and judgmental bias. For the current study, only the subscale assessing interpretation bias was administered. This scale consists of short scripts describing 20 social situations and 4 non-social control situations. The social situations can be divided into positive, ambiguous, mildly negative, and profoundly negative social situations. Each script is followed with four alternative answers reflecting a positive, neutral, negative, or profoundly negative interpretation of the situation. Participants are asked to rank the answers according to their probability. The score used in this study reflects the mean rank of the profoundly negative interpretation ranging from 1 to 4. Other, than described in the study protocol ^[1]^, higher scores reflect more negatively biased processing. Cronbach’s *α* for the IJQ at post-assessment was 0.92.

**Adult Rejection Sensitivity Questionnaire (A-RSQ):** The A-RSQ ^[19]^ is an adapted adult version of the Rejection Sensitivity Questionnaire ^[20]^. Nine hypothetical social situations are presented to the participants, and they are asked to state how they would think or feel in those situations. Respondents first indicate on a 6-point Likert scale how concerned they would be in this situation (very unconcerned – very concerned), afterwards they indicate on a 6-point Likert scale how likely they would expect to be accepted in this situation (very unlikely – very likely). The mean value for rejection sensitivity is calculated by first multiplying the concern response by the expectation response for each item and finally averaging these values by nine. Higher scores indicate a more pronounced sensitivity to rejection. The A-RSQ showed high internal consistency in a study with adults with borderline personality disorder, and acceptable consistency was demonstrated in a sample of healthy adults ^[21]^. In the current sample, Cronbach’s *α* for the IJQ at post-assessment was 0.78.

**Cognitive Behavior Avoidance Scale (CBAS)**: The CBAS ^[22,23]^ is a scale assessing cognitive and behavior avoidance in social and non-social situations. For the current study, we used the 8-item subscale Behavior-social avoidance. The items focus on social behavior and participants are asked to respond on a 5-point Likert scale ranging from 1 (not at all true for me) to 5 (extremely true for me). Higher scores indicate greater behavioral avoidance. Cronbach’s *α* for the CBAS at post-assessment was 0.83.

**Distress Disclosure Index (DDI)**: The DDI ^[24]^ is a 12-item scale assessing the degree to which a person feels comfortable disclosing personally distressing information to others. Items are rated on a 5-point Likert scale ranging from 1 (strongly disagree) to 5 (strongly agree). The DDI has good psychometric properties ^[25]^ and in the current sample, Cronbach’s *α* for the DDI at post-assessment was 0.93.

**Kernis-Goldman Authenticity Inventory – short form (KGAI-SF)**: The KGAI-SF is a 20-item short from of the Kernis-Goldman Authenticity Inventory. The items are rated on a 5-point Likert scale wit response options from 1 (strongly disagree) to 5 (strongly agree). The KGAI-SF shows good psychometric properties and in the current sample, Cronbach’s *α* for the KGAI-SF at post-assessment was 0.84.

**Bern Embitterment Inventory (BVI):** The BVI is a scale to assess embitterment. In this study, we used the 4-item subscale misanthropy to assess the respective construct. The four items are rated on a 5-point Likert scale ranging from 0 (I do not agree) to 4 (I agree). Cronbach’s *α* for the BVI at post-assessment was 0.83.

**Motivation for Solitude Scale – Short form (MSS-SF)**: The MSS-SF is a valid and reliable scale assessing the motivation for solitude ^[26]^. In the current study, we used the 8-item subscale assessing the degree to which individuals engage in solitude for self-determined reasons. Items are rated on a 4-point Likert scale with response options from 1 (not at all important) to 4 (very important). Higher scores indicate a higher degree of self-determined motivation for solitude. Cronbach’s *α* for the MSS-SF at post-assessment was 0.83.

**UCLA Loneliness Scale – 3-item short form (UCLA-3)**: The UCLA-3 ^[27]^ is a 3-item short form derived from the R-UCLA Loneliness Scale ^[28]^. The three items rated on a 5-point Likert scale ranging from 0 (never) to 4 (very often) and the item scores are summed, with higher scores indicating higher levels of loneliness. The German version of the scale shows good psychometric properties and German population norms are available ^[29]^. In the current study, Cronbach’s *α* for the UCLA-3 at post-assessment was 0.77.

**Single item loneliness:** A direct measure of loneliness was administered, since previous studies have revealed discrepancies, e.g., in the prevalence of loneliness, when loneliness was assessed directly or indirectly ^[30]^. We used the single-item “Do you feel lonely”, which was rated on a 4-point scale ranging from 0 (no, never) to 3 (yes, very often).

***Client Satisfaction Questionnaire (CSQ-8):*** The CSQ-8 is a valid 8-item scale assessing client satisfaction with the treatment ^[31]^. The items are rated on a 4-point Likert-scale and the response options range from 1 (low satisfaction) to 4 (high satisfaction). Items are summed up and higher scores indicate greater satisfaction with the treatment. We adapted the wording of the questionnaire to explore participants’ satisfaction with the internet intervention applied in this study. We used the word “online-program” instead of “treatment”. Cronbach’s *α* for the CSQ-8 at post-assessment was 0.89.

***System Usability Scale (SUS):*** The SUS is a 10-item scale to assess the usability of systems ^[32]^. For this trial, we adapted the scale to measure the experienced usability of the internet-based program used in this study. The items are rated on a 5-point Likert scale with response options ranging from 0 (strongly disagree) to 4 (strongly agree). The usability score results from multiplying the sum of all items by 2.5 and ranges from 0 to 100, with higher scores indicating greater experienced usability. Cronbach’s *α* for the SUS at post-assessment was 0.84.

***Inventory for the Assessment of Negative Effects of Psychotherapy (INEP):*** The INEP ^[33]^ is a scale to assess the negative effects of the internet-based program used in this study. This scale assesses any adverse effects on social, intrapersonal, or work-related situations and asks, whether they are attributed to the intervention. A sum score reflects the negative effects across the different life domains. A negative effect exists if a deterioration is indicated in a life area that is attributed to the intervention. Higher sum scores represent more negative effects (range: 0-15). As in other studies, we adapted the questionnaire slightly for its use with internet-based interventions.

***Mini-DIPS Open Access:*** The Mini-DIPS-OA is a openly accessibly structured interview that allows to reliably assess diagnoses according to the DSM-5 and ICD-10 ^[34]^.

# Results

## Baseline Evaluation and Preliminary Analyses

### Table S2

*Differences between groups in primary and secondary outcomes at baseline.*

|  | GU (n = 98) | AM (n = 97) | WL (n = 48) | Statistic |
| --- | --- | --- | --- | --- |
| UCLA-9 | 24.04 (3.18) | 24.67 (3.51) | 24.17 (3.33) | *F* (2,240) = 0.92; *p* = .40 |
| PHQ-9 | 8.90 (3.35) | 8.87 (3.31) | 8.50 (3.24) | *F* (2,240) = 0.26; *p* = .77 |
| SIAS-6 | 5.73 (4.36) | 5.85 (4.73)^a^ | 5.81 (3.93) | *F* (2,239) = 0.02; *p* = .98 |
| SPS-6 | 3.23 (3.28) | 3.42 (4.22)^a^ | 3.25 (3.64) | *F* (2,239) = 0.07; *p* = .94 |
| SNI | 10.63 (5.24) | 11.33 (7.05) | 11.50 (6.40) | *F* (2,240) = 0.44; *p* = .65 |
| RSES | 1.81 (0.65) | 1.74 (0.70)^a^ | 1.69 (0.72) | *F* (2,239) = 0.53; *p* = .59 |
| SWLS | 19.18 (5.93) | 17.85 (5.97) | 17.88 (7.19) | *F* (2,240) = 1.34; *p* = .26 |
| SOCS-S | 3.39 (0.64) | 3.36 (0.66) | 3.42 (0.65) | *F* (2,240) = 0.12; *p* = .88 |
| UCLA-3 | 7.46 (2.03) | 7.74 (2.11) | 7.42 (2.43) | *F* (2,240) = 0.56; *p* = .57 |
| CBAS | 2.44 (0.75) | 2.50 (0.76) | 2.47 (0.80) | *F* (2,240) = 0.20; *p* = .82 |
| IJQ_tot | 1.61 (0.40)^b^ | 1.65 (0.41)^c^ | 1.73 (0.55)^d^ | *F* (2,232) = 1.27; *p* = .28 |
| DDI | 3.26 (0.82)^b^ | 2.96 (0.82) | 3.00 (0.91) | *F* (2,239) = 3.38; *p* = .04 |
| PID5BF+ | 1.03 (0.31) | 1.07 (0.35)^a^ | 1.03 (0.31) | *F* (2,239) = 0.37; *p* = .69 |
| BVI | 1.64 (1.00) | 1.79 (0.99) | 1.68 (0.90) | *F* (2,240) = 0.63; *p* = .53 |
| KGAI-SF | 3.59 (0.53) | 3.49 (0.59) | 3.55 (0.50) | *F* (2,240) = 0.79; *p* = .46 |
| A-RSQ | 11.52 (4.00)^b^ | 11.21 (4.38) | 10.99 (4.34) | *F* (2,239) = 0.28; *p* = .76 |
| MSS-SF | 2.43 (0.64) | 2.36 (0.66) | 2.48 (0.59) | *F* (2,240) = 0.54; *p* = .58 |
| Lonely_dir | 1.88 (0.69) | 1.95 (0.75)^a^ | 1.98 (0.76) | *F* (2,239) = 0.39; *p* = .68 |

*Note.* GU = SOLUS-D with guidance; AM = SOLUS-D with automated message; WL = waitlist control group. UCLA-9 = 9-item version of the UCLA Loneliness Scale; PHQ-9 = 9-item Depression Module of the Patient Health Questionnaire; SIAS-6 = Social Interaction Anxiety Scale; SPS-6 = Social Phobia Scale; SNI = Social Network Index - size of social network ; SWLS: Satisfaction with life; RSES = Rosenberg Self-esteem Scale; SOCS-S = Sussex-Oxford Compassion for the Self Scale; CBAS = Cognitive-Behavioral Avoidance Scale – subscale Behavior-social avoidance ; IJQ_tot = Interpretation and Judgmental Bias Questionnaire – total score; DDI = Distress Disclosure Index; PID5BF+ = Personality Inventory for the DSM-5 Brief Form Plus; BVI = Bern Embitterment Inventory – subscale misanthropy; KGAI-SF = Kernis Goldman Authenticity Inventory - short form ; A-RSQ = Adult-Rejection Sensitivity Questionnaire; MSS-SF = the Motivation for Solitude Scale – Short Form; Lonely_dir = single item to assess loneliness directly (“Do you feel lonely?”).

^a^ n = 96

^b^ n = 97

^c^ n = 91

^d^ n = 47

## Study Dropout Analysis

### Table S3

*Differences between completer and non-completer at baseline in primary and secondary outcomes.*

|  | Completer  (n = 180) | Non-Completer  (n = 63) | Statistic |
| --- | --- | --- | --- |
| UCLA-9 | 24.13 (3.20) | 24.84 (3.71) | *t*(241) = 1.45, *p* = .15 |
| PHQ-9 | 8.74 (3.26) | 9.00 (3.45) | *t*(241) = 0.49, *p* = .62 |
| SIAS-6 | 5.66 (4.13) | 6.21 (5.18)^a^ | *t*(240) = 0.85, *p* = .40 |
| SPS-6 | 3.22 (3.62) | 3.58 (4.07)^a^ | *t*(240) = 0.66, *p* = .51 |
| SNI | 11.05 (6.06) | 11.17 (6.74) | *t*(241) = 0.14, *p* = .89 |
| RSES | 1.78 (0.68) | 1.68 (0.70)^a^ | *t*(240) = -1.05, *p* = .29 |
| SWLS | 18.49 (5.93) | 18.11 (7.02) | *t*(241) = -0.41, *p* = .68 |
| SOCS-S | 3.41 (0.64) | 3.32 (0.67) | *t*(241) = -0.93, *p* = .35 |
| UCLA-3 | 7.51 (2.13) | 7.71 (2.20) | *t*(241) = 0.65, *p* = .52 |
| CBAS | 2.46 (0.73) | 2.49 (0.86) | *t*(241) = 0.24, *p* = .81 |
| IJQ_tot | 1.62 (0.44)^b^ | 1.74 (0.43)^c^ | *t*(233) = 1.88, *p* = .06 |
| DDI | 3.04 (0.81) | 3.22 (0.95)^a^ | *t*(240) = 1.45, *p* = .15 |
| PID5BF+ | 1.03 (0.29) | 1.09 (0.40)^a^ | *t*(240) = 1.40, *p* = .16 |
| BVI | 1.73 (0.95) | 1.64 (1.06) | *t*(241) = -0.64, *p* = .52 |
| KGAI-SF | 3.57 (0.52) | 3.46 (0.63) | *t*(241) = -1.30, *p* = .20 |
| A-RSQ | 11.01 (4.22)^d^ | 12.08 (4.10) | *t*(240) = 1.73, *p* = .08 |
| MSS-SF | 2.41 (0.65) | 2.41 (0.63) | *t*(241) = -0.00, *p* = .99 |
| Lonely_dir | 1.88 (0.71)^d^ | 2.06 (0.76) | *t*(240) = 1.76, *p* = .08 |

*Note.* Completer = Baseline and post-assessment available, Non-Completer = only baseline assessment available. UCLA-9 = 9-item version of the UCLA Loneliness Scale; PHQ-9 = 9-item Depression Module of the Patient Health Questionnaire; SIAS-6 = Social Interaction Anxiety Scale; SPS-6 = Social Phobia Scale; SNI = Social Network Index - size of social network ; SWLS: Satisfaction with life; RSES = Rosenberg Self-esteem Scale; SOCS-S = Sussex-Oxford Compassion for the Self Scale; CBAS = Cognitive-Behavioral Avoidance Scale – subscale Behavior-social avoidance ; IJQ_tot = Interpretation and Judgmental Bias Questionnaire – total score; DDI = Distress Disclosure Index; PID5BF+ = Personality Inventory for the DSM-5 Brief Form Plus; BVI = Bern Embitterment Inventory – subscale misanthropy; KGAI-SF = Kernis Goldman Authenticity Inventory - short form ; A-RSQ = Adult-Rejection Sensitivity Questionnaire; MSS-SF = the Motivation for Solitude Scale – Short Form; Lonely_dir = single item to assess loneliness directly (“Do you feel lonely?”).

^a^ n = 62

^b^ n = 175

^c^ n = 60

^d^ n = 179

### Table S4

*Baseline Characteristics completer vs. non-completer.*

|  | Completer  (n = 180) | Non-Completer  (n = 63) | Statistic |
| --- | --- | --- | --- |
| Mean age, years (SD) | 47.2 (14.5) | 41.6 (15.3) | *t*(241) = -2.62, *p* = .009 |
| Gender, n (%) |  |  | *χ^2^*(2) = 1.19; *p* = .55 |
| Female | 143 (79.4%) | 48 (76.2%) |  |
| Male | 35 (19.4%) | 15 (23.8%) |  |
| Other | 2 (1.1%) | 0 (0.0%) |  |
| Marital status n (%) |  |  | *χ^2^*(1) = 0.05; *p* = .82 |
| Single/divorced/widowed | 136 (75.6%) | 46 (73.0%) |  |
| Married/partnered | 44 (24.4%) | 17 (27.0%) |  |
| Living situation, n (%) |  |  | *χ^2^*(3) = 4.14; *p* = .25 |
| Alone | 120 (66.7%) | 33 (52.4%) |  |
| With partner/family | 33 (18.3%) | 16 (25.4%) |  |
| Shared flat | 18 (10.0%) | 9 (14.3%) |  |
| Other | 9 (5.0%) | 5 (7.9%) |  |
| Highest educational level, n (%) |  |  | *χ^2^*(2) = 1.30; *p* = .52 |
| Middle school | 3 (1.7%) | 2 (3.2%) |  |
| High school/some college | 61 (34.1%) | 25 (39.7%) |  |
| University | 115 (64.2%) | 36 (57.1%) |  |
| Employment, n (%) |  |  | *χ^2^*(5) = 4.02; *p* = .55 |
| Full-time paid work | 62 (34.8%) | 18 (29.0%) |  |
| Part-time paid work | 51 (28.7%) | 21 (33.9%) |  |
| Student/in training | 10 (5.6%) | 5 (8.1%) |  |
| unemployed | 11 (6.2%) | 7 (11.3%) |  |
| Househusband/Housewife | 6 (3.4%) | 2 (3.2%) |  |
| Retired | 38 (21.3%) | 4 (6.5%) |  |
| Current psychological treatment ^a^ | 61 (33.9%) | 18 (28.6%) | *χ^2^*(1) = 0.38; *p* = .53 |
| Current use of psychotropic medication ^a^ | 32 (17.8%) | 8 (12.7%) | *χ^2^*(1) = 0.55; *p* = .46 |
| Mean duration of loneliness, months(SD) | 145.57 (180.28)^c^ | 122.33 (121.83) | *χ^2^*(236) = -0.95; *p* = .34 |
| Psychiatric diagnoses ^b^ |  |  |  |
| Major depressive disorder | 21 (11.7%) | 6 (9.5%) | *χ^2^*(1) = 0.05; *p =* .82 |
| Panic disorder | 14 (7.8%) | 3 (4.8%) | *χ^2^*(1) =0.27; *p =* .60 |
| Agoraphobia | 11 (6.1%) | 3 (4.8%) | *χ^2^*(1) = 0.00; *p =* .94 |
| Social anxiety disorder | 47 (26.1%) | 24 (38.1%) | *χ^2^*(1) = 2.69; *p =* .10 |
| Generalized anxiety disorder | 26 (14.4%) | 12 (19.0%) | *χ^2^*(1) = 0.44; *p =* .51 |
| Obsessive compulsive disorder | 5 (2.8%) | 3 (4.8%) | *χ^2^*(1) = 0.12; *p =* .73 |
| Post-traumatic Stress Disorder | 7 (3.9%) | 3 (4.8%) | *χ^2^*(1) = 0.00; *p =* 1.00 |
| Eating disorder | 6 (2.1%) | 1 (2.1%) | *χ^2^*(1) = 0.08; *p =* .78 |

*Note.* Completer = Baseline and post-assessment are available, Non-Completer = only baseline assessment is available.

^a^ Reflects the number and percentage of participants answering “yes” to this question.

^b^ Reflects the number and percentage of participants fulfilling the respective psychological diagnosis as indicated by the Mini-DIPS during screening.

^c^ n = 175

## Mixed effects models

### Table S5

Mixed effects model with UCLA-9 as outcome

|  | **UCLA-9 Loneliness Scale** | | | | |
| --- | --- | --- | --- | --- | --- |
| Predictors | Estimates | SE | 95% CI | *t*-value | *p* |
| (Intercept) | 24.17 | 0.52 | 23.14 – 25.19 | 46.39 | **<.001** |
| timepoint [2] | -1.05 | 0.48 | -1.99 – -0.11 | -2.19 | **.029** |
| condition [05_automatedEmail_assigned] | 0.50 | 0.64 | -0.75 – 1.76 | 0.79 | .430 |
| condition [05_guidance_assigned] | -0.13 | 0.64 | -1.38 – 1.12 | -0.20 | .843 |
| timepoint [2] × condition [05_automatedEmail_assigned] | -1.69 | 0.62 | -2.91 – -0.48 | -2.73 | **.007** |
| timepoint [2] × condition [05_guidance_assigned] | -2.46 | 0.61 | -3.66 – -1.27 | -4.04 | **<.001** |
| **Random Effects** | | | | | |
| σ^2^ | 5.32 | | | | |
| τ_00_ _id_ | 7.71 | | | | |
| ICC | 0.59 | | | | |
| N _id_ | 243 | | | | |
| Observations | 423 | | | | |
| Marginal *R^2^* / Conditional *R^2^* | 0.144 / 0.651 | | | | |

### Table S6

Mixed effects model with PHQ-9 as outcome

|  | **PHQ-9** | | | | |
| --- | --- | --- | --- | --- | --- |
| Predictors | Estimates | SE | 95% CI | *t*-value | *p* |
| (Intercept) | 8.50 | 0.50 | 7.51 – 9.49 | 16.86 | **<.001** |
| timepoint [2] | -0.35 | 0.54 | -1.41 – 0.70 | -0.66 | .511 |
| condition [05_automatedEmail_assigned] | 0.37 | 0.62 | -0.85 – 1.58 | 0.59 | .553 |
| condition [05_guidance_assigned] | 0.40 | 0.62 | -0.81 – 1.61 | 0.65 | .518 |
| timepoint [2] × condition [05_automatedEmail_assigned] | -1.64 | 0.69 | -3.00 – -0.28 | -2.37 | **.018** |
| timepoint [2] × condition [05_guidance_assigned] | -2.54 | 0.68 | -3.88 – -1.20 | -3.71 | **<.001** |
| **Random Effects** | | | | | |
| σ^2^ | 6.72 | | | | |
| τ_00_ _id_ | 5.48 | | | | |
| ICC | 0.45 | | | | |
| N _id_ | 243 | | | | |
| Observations | 422 | | | | |
| Marginal *R^2^* / Conditional *R^2^* | 0.092 / 0.500 | | | | |

### Table S7

Mixed effects model with SIAS-6 as outcome

|  | **SIAS-6** | | | | |
| --- | --- | --- | --- | --- | --- |
| Predictors | Estimates | SE | 95% CI | *t*-value | *p* |
| (Intercept) | 5.81 | 0.62 | 4.60 – 7.03 | 9.42 | **<.001** |
| timepoint [2] | -0.13 | 0.40 | -0.92 – 0.67 | -0.31 | .755 |
| condition [05_automatedEmail_assigned] | 0.04 | 0.76 | -1.44 – 1.53 | 0.06 | .956 |
| condition [05_guidance_assigned] | -0.08 | 0.75 | -1.56 – 1.40 | -0.10 | .918 |
| timepoint [2] × condition [05_automatedEmail_assigned] | -1.06 | 0.53 | -2.10 – -0.02 | -2.00 | **.046** |
| timepoint [2] × condition [05_guidance_assigned] | -0.97 | 0.52 | -1.99 – 0.05 | -1.86 | .063 |
| **Random Effects** | | | | | |
| σ^2^ | 3.63 | | | | |
| τ_00_ _id_ | 14.64 | | | | |
| ICC | 0.80 | | | | |
| N _id_ | 242 | | | | |
| Observations | 411 | | | | |
| Marginal *R^2^* / Conditional *R^2^* | 0.015 / 0.804 | | | | |

### Table S8

Mixed effects model with SPS-6 as outcome

|  | **SPS-6** | | | | |
| --- | --- | --- | --- | --- | --- |
| Predictors | Estimates | SE | 95% CI | *t*-value | *p* |
| (Intercept) | 3.25 | 0.53 | 2.21 – 4.29 | 6.12 | **<.001** |
| timepoint [2] | 0.68 | 0.37 | -0.05 – 1.41 | 1.84 | .066 |
| condition [05_automatedEmail_assigned] | 0.17 | 0.65 | -1.11 – 1.45 | 0.26 | .798 |
| condition [05_guidance_assigned] | -0.02 | 0.65 | -1.29 – 1.26 | -0.02 | .981 |
| timepoint [2] × condition [05_automatedEmail_assigned] | -1.83 | 0.49 | -2.79 – -0.88 | -3.76 | **<.001** |
| timepoint [2] × condition [05_guidance_assigned] | -1.14 | 0.48 | -2.08 – -0.20 | -2.39 | **.017** |
| **Random Effects** | | | | | |
| σ^2^ | 3.06 | | | | |
| τ_00_ _id_ | 10.48 | | | | |
| ICC | 0.77 | | | | |
| N _id_ | 242 | | | | |
| Observations | 411 | | | | |
| Marginal *R^2^* / Conditional *R^2^* | 0.016 / 0.777 | | | | |

### Table S9

Mixed effects model with SNI as outcome

|  | **SNI** | | | | |
| --- | --- | --- | --- | --- | --- |
| Predictors | Estimates | SE | 95% CI | *t*-value | p |
| (Intercept) | 11.50 | 0.91 | 9.71 – 13.29 | 12.61 | **<.001** |
| timepoint [2] | 0.52 | 0.78 | -1.01 – 2.05 | 0.67 | .501 |
| condition [05_automatedEmail_assigned] | -0.17 | 1.11 | -2.36 – 2.02 | -0.15 | .879 |
| condition [05_guidance_assigned] | -0.87 | 1.11 | -3.06 – 1.32 | -0.78 | .436 |
| timepoint [2] × condition [05_automatedEmail_assigned] | -0.11 | 1.01 | -2.10 – 1.87 | -0.11 | .910 |
| timepoint [2] × condition [05_guidance_assigned] | -0.05 | 0.99 | -2.00 – 1.90 | -0.05 | .961 |
| **Random Effects** | | | | | |
| σ^2^ | 14.04 | | | | |
| τ_00_ _id_ | 25.87 | | | | |
| ICC | 0.65 | | | | |
| N _id_ | 243 | | | | |
| Observations | 423 | | | | |
| Marginal *R^2^* / Conditional *R^2^* | 0.005 / 0.650 | | | | |

### Table S10

Mixed effects model with SNI as outcome

|  | **RSES** | | | | |
| --- | --- | --- | --- | --- | --- |
| Predictors | Estimates | SE | 95% CI | *t*-value | p |
| (Intercept) | 1.69 | 0.09 | 1.50 – 1.87 | 18.00 | **<.001** |
| timepoint [2] | 0.17 | 0.08 | 0.01 – 0.33 | 2.12 | **.035** |
| condition [05_automatedEmail_assigned] | 0.05 | 0.11 | -0.17 – 0.28 | 0.45 | .650 |
| condition [05_guidance_assigned] | 0.12 | 0.11 | -0.11 – 0.34 | 1.04 | .300 |
| timepoint [2] × condition [05_automatedEmail_assigned] | 0.11 | 0.11 | -0.10 – 0.32 | 1.03 | .305 |
| timepoint [2] × condition [05_guidance_assigned] | 0.11 | 0.10 | -0.10 – 0.31 | 1.01 | .313 |
| **Random Effects** | | | | | |
| σ^2^ | 0.15 | | | | |
| τ_00_ _id_ | 0.27 | | | | |
| ICC | 0.65 | | | | |
| N _id_ | 242 | | | | |
| Observations | 411 | | | | |
| Marginal *R^2^* / Conditional *R^2^* | 0.044 / 0.666 | | | | |

### Table S11

Mixed effects model with SWLS as outcome

|  | **SWLS** | | | | |
| --- | --- | --- | --- | --- | --- |
| Predictors | Estimates | SE | 95% CI | *t*-value | *p* |
| (Intercept) | 17.87 | 0.93 | 16.05 – 19.70 | 19.26 | **<.001** |
| timepoint [2] | 0.90 | 0.61 | -0.29 – 2.10 | 1.49 | .138 |
| condition [05_automatedEmail_assigned] | -0.03 | 1.13 | -2.26 – 2.20 | -0.03 | .979 |
| condition [05_guidance_assigned] | 1.31 | 1.13 | -0.92 – 3.54 | 1.15 | .249 |
| timepoint [2] × condition [05_automatedEmail_assigned] | 1.16 | 0.80 | -0.40 – 2.73 | 1.46 | .146 |
| timepoint [2] × condition [05_guidance_assigned] | 0.30 | 0.78 | -1.23 – 1.84 | 0.39 | .697 |
| **Random Effects** | | | | | |
| σ^2^ | 8.18 | | | | |
| τ_00_ _id_ | 33.19 | | | | |
| ICC | 0.80 | | | | |
| N _id_ | 243 | | | | |
| Observations | 412 | | | | |
| Marginal *R^2^* / Conditional *R^2^* | 0.021 / 0.807 | | | | |

### Table S12

Mixed effects model with SOCS-S as outcome

|  | **SOCS-S** | | | | |
| --- | --- | --- | --- | --- | --- |
| Predictors | Estimates | SE | 95% CI | *t*-value | *p* |
| (Intercept) | 3.42 | 0.09 | 3.23 – 3.60 | 36.94 | **<.001** |
| timepoint [2] | -0.05 | 0.08 | -0.20 – 0.10 | -0.64 | .524 |
| condition [05_automatedEmail_assigned] | -0.06 | 0.11 | -0.28 – 0.17 | -0.49 | .622 |
| condition [05_guidance_assigned] | -0.03 | 0.11 | -0.25 – 0.19 | -0.26 | .798 |
| timepoint [2] × condition [05_automatedEmail_assigned] | 0.14 | 0.10 | -0.06 – 0.34 | 1.35 | .177 |
| timepoint [2] × condition [05_guidance_assigned] | 0.31 | 0.10 | 0.12 – 0.51 | 3.11 | **.002** |
| **Random Effects** | | | | | |
| σ^2^ | 0.14 | | | | |
| τ_00_ _id_ | 0.27 | | | | |
| ICC | 0.66 | | | | |
| N _id_ | 243 | | | | |
| Observations | 417 | | | | |
| Marginal *R^2^* / Conditional *R^2^* | 0.024 / 0.666 | | | | |

### Table S13

Mixed effects model with CBAS as outcome

|  | **CBAS** | | | | |
| --- | --- | --- | --- | --- | --- |
| Predictors | Estimates | std. Error | CI | Statistic | p |
| (Intercept) | 2.47 | 0.11 | 2.26 – 2.68 | 23.00 | **<.001** |
| timepoint [2] | 0.13 | 0.07 | -0.02 – 0.28 | 1.73 | .084 |
| condition [05_automatedEmail_assigned] | 0.04 | 0.13 | -0.22 – 0.30 | 0.29 | .772 |
| condition [05_guidance_assigned] | -0.03 | 0.13 | -0.29 – 0.23 | -0.24 | .810 |
| timepoint [2] × condition [05_automatedEmail_assigned] | -0.23 | 0.10 | -0.43 – -0.04 | -2.41 | **.016** |
| timepoint [2] × condition [05_guidance_assigned] | -0.26 | 0.10 | -0.45 – -0.08 | -2.77 | **.006** |
| **Random Effects** | | | | | |
| σ^2^ | 0.12 | | | | |
| τ_00_ _id_ | 0.43 | | | | |
| ICC | 0.78 | | | | |
| N _id_ | 243 | | | | |
| Observations | 413 | | | | |
| Marginal *R^2^* / Conditional *R^2^* | 0.013 / 0.782 | | | | |

### Table S14

Mixed effects model with IJQ as outcome

|  | **IJQ** | | | | |
| --- | --- | --- | --- | --- | --- |
| Predictors | Estimates | SE | 95% CI | *t*-value | *p* |
| (Intercept) | 1.73 | 0.07 | 1.60 – 1.86 | 26.02 | **<.001** |
| timepoint [2] | -0.06 | 0.06 | -0.17 – 0.05 | -1.10 | .271 |
| condition [05_automatedEmail_assigned] | -0.08 | 0.08 | -0.24 – 0.08 | -0.95 | .344 |
| condition [05_guidance_assigned] | -0.12 | 0.08 | -0.28 – 0.04 | -1.50 | .134 |
| timepoint [2] × condition [05_automatedEmail_assigned] | -0.09 | 0.07 | -0.24 – 0.05 | -1.24 | .214 |
| timepoint [2] × condition [05_guidance_assigned] | -0.07 | 0.07 | -0.22 – 0.07 | -1.03 | .305 |
| **Random Effects** | | | | | |
| σ^2^ | 0.07 | | | | |
| τ_00_ _id_ | 0.14 | | | | |
| ICC | 0.66 | | | | |
| N _id_ | 236 | | | | |
| Observations | 396 | | | | |
| Marginal *R^2^* / Conditional *R^2^* | 0.034 / 0.676 | | | | |

### Table S15

Mixed effects model with DDI as outcome

|  | **DDI** | | | | |
| --- | --- | --- | --- | --- | --- |
| Predictors | Estimates | SE | 95% CI | *t*-value | *p* |
| (Intercept) | 3.00 | 0.12 | 2.77 – 3.23 | 25.47 | **<.001** |
| timepoint [2] | 0.04 | 0.07 | -0.10 – 0.18 | 0.55 | .586 |
| condition [05_automatedEmail_assigned] | -0.03 | 0.14 | -0.32 – 0.25 | -0.24 | .814 |
| condition [05_guidance_assigned] | 0.26 | 0.14 | -0.02 – 0.55 | 1.83 | .069 |
| timepoint [2] × condition [05_automatedEmail_assigned] | 0.19 | 0.10 | 0.00 – 0.38 | 1.99 | **.047** |
| timepoint [2] × condition [05_guidance_assigned] | 0.19 | 0.09 | 0.01 – 0.38 | 2.05 | **.041** |
| **Random Effects** | | | | | |
| σ^2^ | 0.12 | | | | |
| τ_00_ _id_ | 0.55 | | | | |
| ICC | 0.82 | | | | |
| N _id_ | 242 | | | | |
| Observations | 412 | | | | |
| Marginal *R^2^* / Conditional *R^2^* | 0.049 / 0.832 | | | | |

### Table S16

Mixed effects model with PID5BF+ as outcome

|  | **PID5BF+** | | | | |
| --- | --- | --- | --- | --- | --- |
| Predictors | Estimates | SE | 95% CI | *t*-value | *p* |
| (Intercept) | 1.03 | 0.05 | 0.94 – 1.13 | 21.48 | **<.001** |
| timepoint [2] | -0.08 | 0.04 | -0.15 – -0.01 | -2.26 | **.024** |
| condition [05_automatedEmail_assigned] | 0.03 | 0.06 | -0.08 – 0.15 | 0.54 | .593 |
| condition [05_guidance_assigned] | -0.01 | 0.06 | -0.12 – 0.11 | -0.12 | .904 |
| timepoint [2] × condition [05_automatedEmail_assigned] | -0.01 | 0.05 | -0.11 – 0.08 | -0.27 | .787 |
| timepoint [2] × condition [05_guidance_assigned] | -0.09 | 0.05 | -0.18 – 0.00 | -1.88 | .061 |
| **Random Effects** | | | | | |
| σ^2^ | 0.03 | | | | |
| τ_00_ _id_ | 0.08 | | | | |
| ICC | 0.74 | | | | |
| N _id_ | 242 | | | | |
| Observations | 410 | | | | |
| Marginal *R^2^* / Conditional *R^2^* | 0.042 / 0.751 | | | | |

### Table S17

Mixed effects model with BVI as outcome

|  | **BVI** | | | | |
| --- | --- | --- | --- | --- | --- |
| Predictors | Estimates | SE | 95% CI | *t*-value | *p* |
| (Intercept) | 1.68 | 0.14 | 1.40 – 1.96 | 11.79 | **<.001** |
| timepoint [2] | 0.02 | 0.09 | -0.16 – 0.19 | 0.18 | .856 |
| condition [05_automatedEmail_assigned] | 0.11 | 0.17 | -0.23 – 0.45 | 0.64 | .523 |
| condition [05_guidance_assigned] | -0.04 | 0.17 | -0.39 – 0.30 | -0.25 | .806 |
| timepoint [2] × condition [05_automatedEmail_assigned] | -0.28 | 0.12 | -0.51 – -0.04 | -2.34 | **.020** |
| timepoint [2] × condition [05_guidance_assigned] | -0.24 | 0.12 | -0.47 – -0.01 | -2.05 | **.041** |
| **Random Effects** | | | | | |
| σ^2^ | 0.18 | | | | |
| τ_00_ _id_ | 0.80 | | | | |
| ICC | 0.82 | | | | |
| N _id_ | 243 | | | | |
| Observations | 412 | | | | |
| Marginal *R^2^* / Conditional *R^2^* | 0.016 / 0.818 | | | | |

### Table S18

Mixed effects model with KGAI-SF as outcome

|  | **KGAI-SF** | | | | |
| --- | --- | --- | --- | --- | --- |
| Predictors | Estimates | SE | 95% CI | *t*-value | *p* |
| (Intercept) | 3.55 | 0.08 | 3.40 – 3.70 | 45.48 | **<.001** |
| timepoint [2] | 0.05 | 0.06 | -0.07 – 0.16 | 0.84 | .402 |
| condition [05_automatedEmail_assigned] | -0.06 | 0.10 | -0.25 – 0.12 | -0.66 | .510 |
| condition [05_guidance_assigned] | 0.03 | 0.10 | -0.15 – 0.22 | 0.37 | .715 |
| timepoint [2] × condition [05_automatedEmail_assigned] | 0.09 | 0.08 | -0.06 – 0.24 | 1.23 | .221 |
| timepoint [2] × condition [05_guidance_assigned] | 0.13 | 0.08 | -0.02 – 0.28 | 1.72 | .086 |
| **Random Effects** | | | | | |
| σ^2^ | 0.08 | | | | |
| τ_00_ _id_ | 0.22 | | | | |
| ICC | 0.74 | | | | |
| N _id_ | 243 | | | | |
| Observations | 413 | | | | |
| Marginal *R^2^* / Conditional *R^2^* | 0.026 / 0.744 | | | | |

### Table S19

Mixed effects model with A-RSQ as outcome

|  | **A-RSQ** | | | | |
| --- | --- | --- | --- | --- | --- |
| Predictors | Estimates | SE | 95% CI | *t*-value | *p* |
| (Intercept) | 10.99 | 0.62 | 9.76 – 12.21 | 17.70 | **<.001** |
| timepoint [2] | 0.38 | 0.46 | -0.53 – 1.29 | 0.82 | .414 |
| condition [05_automatedEmail_assigned] | 0.23 | 0.76 | -1.27 – 1.72 | 0.30 | .766 |
| condition [05_guidance_assigned] | 0.51 | 0.76 | -0.98 – 2.00 | 0.68 | .498 |
| timepoint [2] × condition [05_automatedEmail_assigned] | -1.86 | 0.60 | -3.04 – -0.67 | -3.08 | **.002** |
| timepoint [2] × condition [05_guidance_assigned] | -2.08 | 0.59 | -3.25 – -0.91 | -3.50 | **.001** |
| **Random Effects** | | | | | |
| σ^2^ | 4.74 | | | | |
| τ_00_ _id_ | 13.76 | | | | |
| ICC | 0.74 | | | | |
| N _id_ | 243 | | | | |
| Observations | 412 | | | | |
| Marginal *R^2^* / Conditional *R^2^* | 0.027 / 0.751 | | | | |

### Table S20

Mixed effects model with MSS-SF as outcome

|  | **MSS-SF** | | | | |
| --- | --- | --- | --- | --- | --- |
| Predictors | Estimates | SE | 95% CI | *t*-value | *p* |
| (Intercept) | 2.48 | 0.09 | 2.29 – 2.66 | 26.59 | **<.001** |
| timepoint [2] | 0.07 | 0.08 | -0.08 – 0.22 | 0.89 | .373 |
| condition [05_automatedEmail_assigned] | -0.11 | 0.11 | -0.34 – 0.11 | -1.00 | .320 |
| condition [05_guidance_assigned] | -0.05 | 0.11 | -0.27 – 0.17 | -0.44 | .657 |
| timepoint [2] × condition [05_automatedEmail_assigned] | 0.07 | 0.10 | -0.13 – 0.27 | 0.70 | .484 |
| timepoint [2] × condition [05_guidance_assigned] | 0.19 | 0.10 | -0.00 – 0.38 | 1.92 | .055 |
| **Random Effects** | | | | | |
| σ^2^ | 0.14 | | | | |
| τ_00_ _id_ | 0.28 | | | | |
| ICC | 0.67 | | | | |
| N _id_ | 243 | | | | |
| Observations | 419 | | | | |
| Marginal *R^2^* / Conditional *R^2^* | 0.026 / 0.678 | | | | |

### Table S21

Mixed effects model with Lonely direct as outcome

|  | **Lonely direct** | | | | |
| --- | --- | --- | --- | --- | --- |
| Predictors | Estimates | SE | 95% CI | *t*-value | *p* |
| (Intercept) | 1.98 | 0.10 | 1.78 – 2.18 | 19.78 | **<.001** |
| timepoint [2] | -0.41 | 0.10 | -0.61 – -0.22 | -4.11 | **<.001** |
| condition [05_automatedEmail_assigned] | -0.03 | 0.12 | -0.27 – 0.21 | -0.27 | .788 |
| condition [05_guidance_assigned] | -0.10 | 0.12 | -0.34 – 0.14 | -0.83 | .406 |
| timepoint [2] × condition [05_automatedEmail_assigned] | -0.21 | 0.13 | -0.47 – 0.05 | -1.61 | .109 |
| timepoint [2] × condition [05_guidance_assigned] | -0.30 | 0.13 | -0.55 – -0.04 | -2.31 | **.022** |
| **Random Effects** | | | | | |
| σ^2^ | 0.24 | | | | |
| τ_00_ _id_ | 0.24 | | | | |
| ICC | 0.51 | | | | |
| N _id_ | 243 | | | | |
| Observations | 418 | | | | |
| Marginal *R^2^* / Conditional *R^2^* | 0.171 / 0.594 | | | | |

## Contrast Analyses Primary and Secondary Outcomes

### Table S22

Estimates, t- values, and p-values of contrasts at post-assessment for measures with significant overall effects in the mixed-effects models.

| Measure | Contrast | Estimate | t-value | p-value |
| --- | --- | --- | --- | --- |
| UCLA-9 | WL vs. INT | 1.8906246 | 3.125 | .0019 |
|  | AM vs. GU | 0.7001642 | 2.376 | .0180 |
| PHQ-9 | WL vs. INT | 1.7086124 | 2.891 | .0041 |
|  | AM vs. GU | 0.4332289 | 1.476 | .1407 |
| SPS-6 | WL vs. INT | 1.4110921 | 2.296 | .0224 |
|  | AM vs. GU | -0.2547624 | -0.871 | .3841 |
| SOCS-S | WL vs. INT | -0.18342278 | -1.703 | .0896 |
|  | AM vs. GU | -0.10067503 | -1.928 | .0546 |
| CBAS | WL vs. INT | 0.24628947 | 1.981 | .0485 |
|  | AM vs. GU | 0.04960185 | 0.846 | .3978 |
| BVI | WL vs. INT | 0.22316203 | 1.360 | .1750 |
|  | AM vs. GU | 0.05771681 | 0.747 | .4553 |
| A-RSQ | WL vs. INT | 1.5966016 | 2.215 | .0275 |
|  | AM vs. GU | -0.0325974 | -0.095 | .9246 |

*Note.* UCLA-9 = 9-item version of the UCLA Loneliness Scale; PHQ-9 = 9-item Depression Module of the Patient Health Questionnaire; SPS-6 = Social Phobia Scale; SOCS-S = Sussex-Oxford Compassion for the Self Scale; CBAS = Cognitive-Behavioral Avoidance Scale – subscale Behavior-social avoidance; BVI = Bern Embitterment Inventory – subscale misanthropy; A-RSQ = Adult-Rejection Sensitivity Questionnaire. GU = SOLUS-D with guidance; AM = SOLUS-D with automated message; WL = waitlist control group; INT = SOLUS-D with guidance and SOLUS-D with automated message pooled together.

## Sensitivity Analyses

### Table S23

Sensitivity Analyses. Observed and estimated means for the primary measure, overall effects, within-group and between-group effects, and post-treatment contrasts.

| Measure | Baseline | |  | Post (observed) | |  | Post (estimated) | |  | Pre -post within-group effect sizes (estimated means)) |  | Overall effects (Time × Group interaction) |  | Contrasts  (at post-assessment) |  | Between-group effect sizes at post-treatment (estimated means) |
| --- | --- | --- | --- | --- | --- | --- | --- | --- | --- | --- | --- | --- | --- | --- | --- | --- |
|  | Mean (SD) | n |  | Mean (SD) | n |  | Mean (SE) | n |  | Cohen’s d [95% CI] |  | F and *df* |  |  |  | Cohen’s d  [95% CI] |
| **a) Per Protocol** |  |  |  |  |  |  |  |  |  |  |  |  |  |  |  |  |
| GU | 23.81 (3.00) | 69 |  | 20.33 (3.71) | 69 |  | 20.33(0.44) | 69 |  | 1.03 [0.67; 1.38] |  | F_(2, 169)_ = 8.27  *p* < .001 |  | WL vs. INT: *p* = .002  GU vs. AM: *p* = .03 |  | GU vs. WL: -0.86 [-1.25; -0.47] |
| AM | 24.68 (3.34) | 57 |  | 21.77 (4.07) | 57 |  | 21.77 (0.49) | 57 |  | 0.78 [0.40; 1.16] |  |  |  |  |  | GU vs. AM: -0.46 [-0.81; -0.10] |
| WL | 24.04 (3.35) | 46 |  | 23.04 (4.10) | 46 |  | 23.04 (0.54) | 46 |  | 0.27 [-0.15; 0.68] |  |  |  |  |  | AM vs. WL: -0.38 [-0.77; 0.01] |
| **b) Psychological Disorder** |  |  |  |  |  |  |  |  |  |  |  |  |  |  |  |  |
| GU | 24.84 (2.73) | 51 |  | 20.83 (3.43) | 36 |  | 20.98 (0.53) | 51 |  | 1.25 [0.82; 1.66] |  | F_(2,98.111)_ = 7.69  *p* < .001 |  | WL vs. INT: *p* = .001  GU vs. AM: *p* = .06 |  | GU vs. WL: -1.16 [-1.66; -0.62] |
| AM | 25.58 (3.14) | 50 |  | 22.00 (3.47) | 32 |  | 22.44 (0.56) | 50 |  | 0.95 [0.53; 1.35] |  |  |  |  |  | GU vs. AM: -0.50 [-0.89; -0.10] |
| WL | 25.17 (3.66) | 24 |  | 24.43 (4.11) | 23 |  | 24.50 (0.69) | 24 |  | 0.17 [-0.40; 0.73] |  |  |  |  |  | AM vs. WL: -0.62 [-1.11; -0.12] |
| **c) In Treatment** |  |  |  |  |  |  |  |  |  |  |  |  |  |  |  |  |
| GU | 24.48 (3.34) | 31 |  | 20.42 (3.66) | 24 |  | 20.75 (0.64) | 31 |  | 1.09 [0.54; 1.61] |  | F_(2,62.238)_ = 5.26  *p* = .008 |  | WL vs. INT: *p* = .06  GU vs. AM: *p* = .01 |  | GU vs. WL: -0.97 [-1.60; -0.31] |
| AM | 25.27 (3.30) | 33 |  | 22.77 (3.48) | 22 |  | 23.23 (0.65) | 33 |  | 0.59 [0.09; 1.08] |  |  |  |  |  | GU vs. AM: -0.75 [-1.25; -0.22] |
| WL | 23.80 (2.93) | 15 |  | 23.87 (4.14) | 15 |  | 23.87 (0.84) | 15 |  | 0.01 [-0.71; 0.73] |  |  |  |  |  | AM vs. WL: -0.20 [-0.81; 0.41] |

*Note*: Per protocol = participants who completed baseline and post-assessment and accessed at least four modules; Psychological Disorder = participants who fulfilled at least one psychological disorder as indicated by the MINI-DIPS interview conducted before randomization; In Treatment = participants attending psychological treatment at baseline. GU = SOLUS-D with guidance; AM = SOLUS-D with automated message; WL = waitlist control group, INT = SOLUS-D with guidance and SOLUS-D with automated message pooled together.

### Table S24

Estimates, *t*- values, *p*-values, and effect sizes (Cohen’s *d*) with 95% CI of contrasts at post-assessment for the sensitivity analyses with significant overall effects in the mixed-effects models with the UCLA-9 as primary outcome.

| Measure | Contrast | Estimate | *t*-value | *p*-value | Cohen’s *d* [95% CI] |
| --- | --- | --- | --- | --- | --- |
| Per Protocol | WL vs. INT | 1.9908467 | 3.214 | .0015 | 0.64 [0.29; 0.98] |
|  | AM vs. GU | 0.7192982 | 2.238 | .0261 | 0.46 [0.10; 0.81] |
| Psychological Disorder | WL vs. INT | 2.7962473 | 3.548 | .0005 | 0.91 [0.45; 1.37] |
|  | AM vs. GU | 0.7305614 | 1.903 | .0585 | 0.50 [0.10; 0.89] |
| In Treatment | WL vs. INT | 1.8806521 | 1.862 | .0649 | 0.60 [0.02; 1.16] |
|  | AM vs. GU | 1.2382746 | 2.520 | .0129 | 0.75 [0.22; 1.25] |

*Note*. UCLA-9 = 9-item version of the UCLA Loneliness Scale. Per protocol = participants who completed baseline and post-assessment and accessed at least four modules; Psychological Disorder = participants who fulfilled at least one psychological disorder as indicated by the MINI-DIPS interview conducted before randomization; In Treatment = participants attending psychological treatment at baseline. GU = SOLUS-D with guidance; AM = SOLUS-D with automated message; WL = waitlist control group, INT = SOLUS-D with guidance and SOLUS-D with automated message pooled together.

### Table S25

Mixed effects model with the UCLA-9 Loneliness Scale as outcome in the Per Protocol Sample

|  | **UCLA-9** | | | | |
| --- | --- | --- | --- | --- | --- |
| Predictors | Estimates | SE | 95% CI | *t*-value | *p* |
| (Intercept) | 24.04 | 0.53 | 23.00 – 25.09 | 45.40 | **<.001** |
| timepoint [2] | -1.00 | 0.48 | -1.95 – -0.05 | -2.08 | **.039** |
| condition [05_automatedEmail_assigned] | 0.64 | 0.71 | -0.76 – 2.04 | 0.90 | .369 |
| condition [05_guidance_assigned] | -0.23 | 0.68 | -1.58 – 1.11 | -0.34 | .735 |
| timepoint [2] × condition [05_automatedEmail_assigned] | -1.91 | 0.65 | -3.19 – -0.64 | -2.95 | **.003** |
| timepoint [2] × condition [05_guidance_assigned] | -2.48 | 0.62 | -3.70 – -1.26 | -3.99 | **<.001** |
| **Random Effects** | | | | | |
| σ^2^ | 5.33 | | | | |
| τ_00_ _id_ | 7.57 | | | | |
| ICC | 0.59 | | | | |
| N _id_ | 172 | | | | |
| Observations | 344 | | | | |
| Marginal *R^2^* / Conditional *R^2^* | 0.157 / 0.652 | | | | |

### Table S26

Mixed effects model with the UCLA-9 Loneliness Scale as outcome in the sample of participants fulfilling the symptoms of at least one psychological diagnosis

|  | **UCLA-9** | | | | |
| --- | --- | --- | --- | --- | --- |
| Predictors | Estimates | SE | 95% CI | *t*-value | *p* |
| (Intercept) | 25.17 | 0.68 | 23.83 – 26.50 | 37.12 | **<.001** |
| timepoint [2] | -0.66 | 0.66 | -1.96 – 0.63 | -1.01 | .313 |
| condition [05_automatedEmail_assigned] | 0.41 | 0.82 | -1.21 – 2.04 | 0.50 | .617 |
| condition [05_guidance_assigned] | -0.32 | 0.82 | -1.94 – 1.30 | -0.39 | .694 |
| timepoint [2] × condition [05_automatedEmail_assigned] | -2.48 | 0.85 | -4.15 – -0.81 | -2.92 | **.004** |
| timepoint [2] × condition [05_guidance_assigned] | -3.20 | 0.83 | -4.84 – -1.57 | -3.85 | **<.001** |
| **Random Effects** | | | | | |
| σ^2^ | 5.00 | | | | |
| τ_00_ _id_ | 6.03 | | | | |
| ICC | 0.55 | | | | |
| N _id_ | 125 | | | | |
| Observations | 216 | | | | |
| Marginal *R^2^* / Conditional *R^2^* | 0.204 / 0.639 | | | | |

### Table S27

Mixed effects model with the UCLA-9 Loneliness Scale as outcome in the sample consisting of participants who are in concurrent psychological treatment

|  | **UCLA-9** | | | | |
| --- | --- | --- | --- | --- | --- |
| Predictors | Estimates | SE | 95% CI | *t*-value | *p* |
| (Intercept) | 23.91 | 0.88 | 22.17 – 25.65 | 27.13 | **<.001** |
| timepoint [2] | -0.04 | 0.93 | -1.87 – 1.79 | -0.04 | .969 |
| condition [05_automatedEmail_assigned] | 1.33 | 1.07 | -0.78 – 3.44 | 1.25 | .214 |
| condition [05_guidance_assigned] | 0.66 | 1.07 | -1.46 – 2.78 | 0.62 | .540 |
| timepoint [2] × condition [05_automatedEmail_assigned] | -1.97 | 1.17 | -4.29 – 0.34 | -1.69 | .094 |
| timepoint [2] × condition [05_guidance_assigned] | -3.78 | 1.17 | -6.10 – -1.46 | -3.22 | **.002** |
| **Random Effects** | | | | | |
| σ^2^ | 6.00 | | | | |
| τ_00_ _id_ | 6.00 | | | | |
| ICC | 0.50 | | | | |
| N _id_ | 87 | | | | |
| Observations | 140 | | | | |
| Marginal *R^2^* / Conditional *R^2^* | 0.159 / 0.580 | | | | |

## References

1 Seewer, N. *et al.* Evaluating the efficacy of a guided and unguided Internet-based self-help intervention for chronic loneliness: Protocol for a 3-arm randomized controlled trial. *JMIR Res Protoc* **11**, e36358, doi:10.2196/36358 (2022).

2 *Creative Commons. Attribution 4.0 International (CC BY 4.0)*, Retrieved 2023/05/22 from https://creativecommons.org/licenses/by/4.0/.

3 Kroenke, K., Spitzer, R. L. & Williams, J. B. The PHQ-9: validity of a brief depression severity measure. *Journal of General Internal Medicine* **16**, 606-613, doi:10.1046/j.1525-1497.2001.016009606.x (2001).

4 Löwe, B., Kroenke, K., Herzog, W. & Gräfe, K. Measuring depression outcome with a brief self-report instrument: sensitivity to change of the Patient Health Questionnaire (PHQ-9). *J Affect Disord* **81**, 61-66, doi:10.1016/S0165-0327(03)00198-8 (2004).

5 Peters, L., Sunderland, M., Andrews, G., Rapee, R. M. & Mattick, R. P. Development of a short form Social Interaction Anxiety (SIAS) and Social Phobia Scale (SPS) using nonparametric item response theory: the SIAS-6 and the SPS-6. *Psychol Assess* **24**, 66-76, doi:10.1037/a0024544 (2012).

6 Diener, E., Emmons, R. A., Larsen, R. J. & Griffin, S. The Satisfaction With Life Scale. *J Pers Assess* **49**, 71-75, doi:10.1207/s15327752jpa4901_13 (1985).

7 Schumacher, J. in *Diagnostische Verfahren zu Lebensqualität und Wohlbefinden* (eds J. Schumacher, A. Klaiberg, & E. Brähler) 305-309 (Hogrefe, 2003).

8 Hinz, A. *et al.* Psychometric properties of the Satisfaction with Life Scale (SWLS), derived from a large German community sample. *Qual Life Res* **27**, 1661-1670, doi:10.1007/s11136-018-1844-1 (2018).

9 von Collani, G. & Herzberg, P. Y. Eine revidierte Fassung der deutschsprachigen Skala zum Selbstwertgefühl von Rosenberg. *Zeitschrift für Differentielle und Diagnostische Psychologie* **24**, 3-7, doi:10.1024//0170-1789.24.1.3 (2003).

10 Rosenberg, M. *Society and the Adolescent Self-Image*. (Princeton University Press, 1965).

11 Gu, J., Baer, R., Cavanagh, K., Kuyken, W. & Strauss, C. Development and Psychometric Properties of the Sussex-Oxford Compassion Scales (SOCS). *Assessment* **27**, 3-20, doi:10.1177/1073191119860911 (2020).

12 Cohen, S., Doyle, W. J., Skoner, D. P., Rabin, B. S. & Gwaltney, J. M. Social ties and susceptibility to the common cold. *Jama* **277**, 1940-1944 (1997).

13 Liebke, L. *et al.* Loneliness, social networks, and social functioning in borderline personality disorder. *Personal Disord* **8**, 349-356, doi:10.1037/per0000208 (2017).

14 Kerber, A. *et al.* Development of a short and ICD-11 compatible measure for DSM-5 maladaptive personality traits using Ant Colony Optimization Algorithms. *Assessment* **29**, 467-487, doi:10.1177/1073191120971848 (2022).

15 Krueger, R. F., Derringer, J., Markon, K. E., Watson, D. & Skodol, A. E. Initial construction of a maladaptive personality trait model and inventory for DSM-5. *Psychol Med* **42**, 1879-1890, doi:10.1017/S0033291711002674 (2012).

16 Zimmermann, J. *et al.* A Common Metric for Self-Reported Severity of Personality Disorder. *Psychopathology* **53**, 168-178, doi:10.1159/000507377 (2020).

17 Brettschneider, M., Neumann, P., Berger, T., Renneberg, B. & Boettcher, J. Internet-based interpretation bias modification for social anxiety: A pilot study. *J Behav Ther Exp Psychiatry* **49**, 21-29, doi:10.1016/j.jbtep.2015.04.008 (2015).

18 Voncken, M. J., Bogels, S. M. & de Vries, K. Interpretation and judgmental biases in social phobia. *Behav Res Ther* **41**, 1481-1488, doi:10.1016/s0005-7967(03)00143-8 (2003).

19 Berenson, K. R. *et al.* Rejection sensitivity and disruption of attention by social threat cues. *J Res Pers* **43**, 1064-1072, doi:10.1016/j.jrp.2009.07.007 (2009).

20 Downey, G. & Feldman, S. I. Implications of rejection sensitivity for intimate relationships. *J Pers Soc Psychol* **70**, 1327-1343, doi:10.1037//0022-3514.70.6.1327 (1996).

21 Bungert, M. *et al.* Rejection sensitivity and symptom severity in patients with borderline personality disorder: effects of childhood maltreatment and self-esteem. *Borderline Personal Disord Emot Dysregul* **2**, 4, doi:10.1186/s40479-015-0025-x (2015).

22 Röthlin, P. *et al.* Vermeidung und Depression. *Diagnostica* **56**, 46-55, doi:10.1026/0012-1924/a000008 (2010).

23 Ottenbreit, N. D. & Dobson, K. S. Avoidance and depression: the construction of the Cognitive–Behavioral Avoidance Scale. *Behaviour Research and Therapy* **42**, 293-313, doi:10.1016/s0005-7967(03)00140-2 (2004).

24 Kahn, J. H. & Hessling, R. M. Measuring the tendency to conceal versus disclose psychological distress. *Journal of Social and Clinical Psychology* **20**, 41-65, doi:10.1521/jscp.20.1.41.22254 (2001).

25 Kahn, J. H., Hucke, B. E., Bradley, A. M., Glinski, A. J. & Malak, B. L. The Distress Disclosure Index: a research review and multitrait-multimethod examination. *J Couns Psychol* **59**, 134-149, doi:10.1037/a0025716 (2012).

26 Thomas, V. & Azmitia, M. Motivation matters: Development and validation of the Motivation for Solitude Scale - Short Form (MSS-SF). *J Adolesc* **70**, 33-42, doi:10.1016/j.adolescence.2018.11.004 (2019).

27 Hughes, M. E., Waite, L. J., Hawkley, L. C. & Cacioppo, J. T. A short scale for measuring loneliness in large surveys: Results from two population-based studies. *Res Aging* **26**, 655-672, doi:10.1177/0164027504268574 (2004).

28 Russel, D., Peplau, L. A. & Cutrona, C. E. The Revised UCLA Loneliness Scale: Concurrent and Discriminant Validity Evidence. *Journal of Personality and Social Psychology* **39**, 472-480, doi:10.1037/0022-3514.39.3.472 (1980).

29 Klein, E. M. *et al.* Loneliness and its relation to mental health in the general population: Validation and norm values of a brief measure. *Journal of Affective Disorders Reports* **4**, 100120, doi:10.1016/j.jadr.2021.100120 (2021).

30 Shiovitz-Ezra, S. & Ayalon, L. Use of direct versus indirect approaches to measure loneliness in later life. *Research on Aging* **34**, 572-591, doi:10.1177/0164027511423258 (2012).

31 Schmidt, J., Lamprecht, F. & Wittmann, W. Zufriedenheit mit der stationären Versorgung. Entwicklung eines Fragebogens und erste Validitätsuntersuchungen. *PPmP: Psychotherapie Psychosomatik Medizinische Psychologie* **39**, 248-255 (1989).

32 Bangor, A., Kortum, P. T. & Miller, J. T. An empirical evaluation of the System Usability Scale. *International Journal of Human-Computer Interaction* **24**, 574-594, doi:10.1080/10447310802205776 (2008).

33 Ladwig, I., Rief, W. & Nestoriuc, Y. Welche Risiken und Nebenwirkungen hat Psychotherapie? - Entwicklung des Inventars zur Erfassung Negativer Effekte von Psychotherapie (INEP). *Verhaltenstherapie* **24**, 252-263, doi:10.1159/000367928 (2014).

34 Margraf, J., Cwik, J. C., Pflug, V. & Schneider, S. Strukturierte klinische Interviews zur Erfassung psychischer Störungen über die Lebensspanne. *Zeitschrift für Klinische Psychologie und Psychotherapie* **46**, 176-186, doi:10.1026/1616-3443/a000430 (2017).
